# Chromosome structures: reduction of certain problems with unequal gene content and gene paralogs to integer linear programming

**DOI:** 10.1186/s12859-017-1944-x

**Published:** 2017-12-06

**Authors:** Vassily Lyubetsky, Roman Gershgorin, Konstantin Gorbunov

**Affiliations:** 1 Institute for Information Transmission Problems of the Russian Academy of Sciences (Kharkevich Institute), Bolshoy Karetny per. 19, build.1, Moscow, 127051 Russia; 2 Faculty of Mechanics and Mathematics, Lomonosov Moscow State University, Leninskiye Gory 1, Main Building, Moscow, 119991 Russia

**Keywords:** Chromosome structure, Chromosomal rearrangement, Ancestral genome, Evolution along the tree, Reconstruction of ancestral genomes, Transformation of chromosome structures, Parsimony principle, Integer linear programming, Efficient algorithms

## Abstract

**Background:**

Chromosome structure is a very limited model of the genome including the information about its chromosomes such as their linear or circular organization, the order of genes on them, and the DNA strand encoding a gene. Gene lengths, nucleotide composition, and intergenic regions are ignored. Although highly incomplete, such structure can be used in many cases, e.g., to reconstruct phylogeny and evolutionary events, to identify gene synteny, regulatory elements and promoters (considering highly conserved elements), etc. Three problems are considered; all assume unequal gene content and the presence of gene paralogs. The distance problem is to determine the minimum number of operations required to transform one chromosome structure into another and the corresponding transformation itself including the identification of paralogs in two structures. We use the DCJ model which is one of the most studied combinatorial rearrangement models. Double-, sesqui-, and single-operations as well as deletion and insertion of a chromosome region are considered in the model; the single ones comprise cut and join. In the reconstruction problem, a phylogenetic tree with chromosome structures in the leaves is given. It is necessary to assign the structures to inner nodes of the tree to minimize the sum of distances between terminal structures of each edge and to identify the mutual paralogs in a fairly large set of structures. A linear algorithm is known for the distance problem without paralogs, while the presence of paralogs makes it NP-hard. If paralogs are allowed but the insertion and deletion operations are missing (and special constraints are imposed), the reduction of the distance problem to integer linear programming is known. Apparently, the reconstruction problem is NP-hard even in the absence of paralogs. The problem of contigs is to find the optimal arrangements for each given set of contigs, which also includes the mutual identification of paralogs.

**Results:**

We proved that these problems can be reduced to integer linear programming formulations, which allows an algorithm to redefine the problems to implement a very special case of the integer linear programming tool. The results were tested on synthetic and biological samples.

**Conclusions:**

Three well-known problems were reduced to a very special case of integer linear programming, which is a new method of their solutions. Integer linear programming is clearly among the main computational methods and, as generally accepted, is fast on average; in particular, computation systems specifically targeted at it are available. The challenges are to reduce the size of the corresponding integer linear programming formulations and to incorporate a more detailed biological concept in our model of the reconstruction.

## Background

### Introduction

Chromosome structure is a large-scale view on the genome; it can be considered as a very limited model of the genome taking into account only the mutual arrangement of genes (ignoring their length and nucleotide composition) on both DNA strands as well as the chromosome type (linear or circular), including gene names (identifiers) [1, 2]. Instead of the term “chromosome structure”, the terms “genome” or even “genotype” are used sometimes [3–5].We prefer the term “chromosome structure”, [6], to outline the distinction between the genome as a biological notion and the considered model. Below we consider the DCJ model widely used in studies of this kind, e.g., [3, 7]. The model includes *standard* DCJ operations: *double*-, *sesqui*-, and *single-*operations; the last ones comprise cut and join operations. They were proposed in [7] and later studied in dozens of publications, for example, in [8–10] where a detailed review of the results and further references are given. The biological mechanisms of the operations are described, e.g., in ([10], chapter 5). Two structures have *equal gene content* if they have no paralogs and contain the same set of names. In the case of unequal gene content, structures can have paralogs, and *supplementary* operations are considered: deletion and insertion of a chromosome connected region [4, 11]; these operations were actively studied, e.g., in [4, 8, 12] where further references are given. The popularity of this model stems from the simplicity and elegance of the underlying mathematical constructs as well as from the ability to model many types of genomic rearrangements. Although highly incomplete, such model can be used in many cases, e.g., to reconstruct phylogeny and evolutionary events, to identify gene synteny, regulatory elements and promoters (considering highly conserved elements), etc.; e.g., ref. to [10, 13]. Remind that paralogs are duplicated genes in the same genome, and the problem of their identification in different genomes is hard and important. The role of the structures with paralogs were described in detail, e.g., in [5, 14, 15].

In the context of chromosome structures, three well-known problems are considered. They are formally described in sections 1.3 and 4.1; here their concepts are introduced together with the corresponding references. The *distance* problem determines the distance between two chromosome structures, i.e., the minimum number of operations required to transform one chromosome structure into another, and the corresponding minimum transformation. Paralogs should be identified so that the resulting structures considered as structures without paralogs have the minimum distance. It is easy to prove that the allowance for paralogs makes the distance problem NP-hard.

A linear-time algorithm was proposed for the distance problem in the absence of paralogs for both equal [3] and unequal [4, 16] gene content. This problem is reduced to integer linear programming formulation (ILP) in [5, 14, 15], where its definition was considerably simplified; specifically, balanced gene content in [5], structure reduction to equal gene content by elimination of unwanted regions with paralogs in [14], and ignoring paralogous genes in [15]. More precisely, in [15] such structures can have paralogs, but after the identification of paralogs, the genes present in one out of both structures (which is a real-life situation) are eliminated and not considered later, which does not seem to be justified in any way. Balanced gene content means the same set of names but with possible paralogs.

In the *reconstruction* problem a phylogenetic tree with chromosome structures in the leaves is given. It is required to assign structures to inner nodes of the tree to minimize the *total distance* between terminal structures of each edge. Thus it can be called a small phylogeny problem; the term “reconstruction” is widely used, e.g., in [13]. As previously, unequal gene content and paralogs in all nodes are allowed. Paralogs should be identified such that the total distance for all resulting structures without paralogs is minimum. It is easy to prove that this problem is NP-hard even in the absence of paralogs. Only heuristic algorithms are known for the problem, among which the algorithms in [6, 13, 17] should be noted. These as well as other publications mentioned above present numerous relevant references; it allows us to avoid detailed historical review here due to publication size limitations.

Thus, *exact* algorithms presented here solve two above problems by reducing them to ILPs. Let us recall that an algorithm is called exact if it is mathematically proved that it always results in a global minimum (hereafter, *minimum point*) of the minimized function involved in the problem statement. The significance of this reduction stems from the appearance of fast methods solving ILP tasks in recent 20 years (e.g., [18, 19]). Note, many combinatorial problems (possibly including ILP) have low complexity on average but can be pretty hard in some special cases. For example, hard inputs are rare for the simplex algorithm for linear programming [20, 21]. Another example, a simple algorithm for solving almost all instances of the famous set partition problem, that is NP-hard, is also proposed in [22].

Finally, the computation of the distance between two chromosome structures with paralogs was reduced to ILP for circular chromosomes in [17]. Here, we define such reduction for arbitrary structures with unequal gene content and paralogs as well as for the reconstruction of such structures along the phylogenetic tree. The computation of a sequence of operations (for the minimum transformation) was also considered previously, e.g., in [16, 17, 23, 24]. An algorithm with a linear complexity solving the distance problem without paralogs and with preset weights of operations (which minimizes the total weight of sequence of operations) that is not based on reduction to ILP was obtained in [23, 24] as well as in our study prepared for publication.

The statement of the *contig* problem is given separately in section 4.1 after the first two problems are clarified.

### Definitions of notions

The definitions relevant to the distance problem can be found in publications in different modifications or the problem can have no strict definition at all. Accordingly, we will briefly review the relevant definitions.

*Chromosome structure* is defined as a directed graph composed of non-intersecting paths (of nonzero length) and cycles (including loops). Loops correspond to circular chromosomes comprising a single gene. Each graph edge represents a gene with no account of its length, and the edge is given the *name* of this gene. The edge direction shows the gene transcription direction. Two extremities of neighboring genes are combined (or *merged*) into a graph node.

In this context, an edge with an assigned name is referred to as a *gene*, while a path or cycle is referred to as a *chromosome*. Repeated names can occur in a structure, they correspond to paralogous genes distinguished by the index *j*: paralogous genes with name *k* get *full names* of the form *k.j*. Full names are unique; a structure with full names only has no paralogs.

Let *adjacency* denote a pair of merged gene extremities, a node of degree 2 in a structure. Here, the extremity is a 5′- or 3′-end of a gene considering that the term “end” is linked to ends of graph edges.

Hereafter, *a* and *b* denote two chromosome structures; *a* is meant to be transformed into *b*. A gene present in both *a* and *b* is referred to as a *common* gene; a gene present in only one structure *a* or *b*, a *special* gene; accordingly, there are *a*- and *b*-special genes. In the case of unequal gene content, two *supplementary* operations can be applied to a structure in addition to the *standard* ones mentioned above: *deletion* and *insertion*. The former is the removal of a connected region of *a*-special genes together with its extremities. Such region can be removed from a circular or linear chromosome (cycle or path); the whole chromosome can be removed as well. If the removed region has neighboring genes on both sides, their extremities are merged. The latter operation, inversely, inserts a connected region of *b*-special genes; in this case, a chromosome is cut in a node and pairs of the new free ends are merged. More precisely, the region can be inserted into or to a boundary of a chromosome or form a new circular or linear chromosome (cycle or path).

Let us recall the notion of common graph *a* + *b* for two structures *a* and *b* given in [17] for *unequal gene content without paralogs*. For equal gene content, such graph was first defined in [25] as the breakpoint graph. For unequal gene content without paralogs, a similar graph was first defined in [12] under the same name. Following [12, 25], *a* + *b* will be referred as the breakpoint graph here. Thus, it is an undirected graph without loops whose nodes are *conventional*, i.e., the extremities of common genes with their names (e.g., 3_*h*_ or 3_*t*_), and *special*, i.e., any maximal by inclusion connected regions of *a*-special or *b*-special genes. The latter are referred to as *blocks*. A block belongs to one of the structures *a* or *b*, and the special node corresponding to it is called an *a*- or a *b*-node, and a set (more precisely, a sequence) of gene names corresponding the block is assigned to it; the latter serves as the special node name. The breakpoint graph edges are as follows. A *conventional* edge connects two conventional nodes if the extremities corresponding to them are merged in *a* or *b*; a *special* edge connects a conventional node to a special one if the extremity corresponding to a conventional node is merged in *a* or *b* with the boundary of the block corresponding to the special node. Double conventional edges are also possible here. A *loop* in *a* + *b* corresponds to a cycle that is a block; stated differently, a special node of this block is connected to itself. A special edge incident to a special node of degree 1 is referred to as a *hanging* edge.

In any case, the breakpoint graph is undirected and includes non-intersecting connected components: paths including isolated nodes and cycles including loops. Non-hanging special edges occur in it in pairs as edges incident to the same special node; it is convenient to consider such pairs as a double edge; subject to this provision, the alternation of *a*- and *b*-edges is preserved. Accordingly, the component *size* is the quantity of conventional edges in it plus half the quantity of special non-hanging edges. The size of isolated conventional nodes and loops equals 0, while that for isolated special nodes equals −1.

A breakpoint graph is considered *final* (or of the *final form*) if all its components are conventional nodes, or cycles without special edges of size (or length) 2, one edge from *a* and the other from *b*. If the *a*, *b*, *c* marks are neglected, the *final* graph *a* + *b* has the form *c* + *c* for a certain structure *c*.

**Fig. 1:**
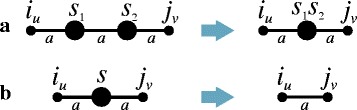
**a** Concatenation of any two neighboring special nodes *s*_1_ and *s*_2_(both from **a**). The nodes *s*_1_ and *s*_2_ are replaced with one special node *s*_1_ *s*_2_ (the concatenated sequence of the sequences of two initial special nodes). Similarly for (**b**). **b** Removal of a special node. Large point is an *a*-special node *s* and the resulting combined edge is marked (**a**). Similarly for (**b**)

Four *standard* operations are allowed on a breakpoint graph, they correspond to the standard operations on a structure. Let us describe them in brief (for details see [16, 17, 23]). *Double-cut-and-paste* is the removal of two edges with the same label (e.g., *a*) and joining four resulting free ends in a new way by two edges with the same label. If this gives rise to an edge with two special nodes (both of which pertaining to either *a* or *b*), it is replaced with one special node to which the concatenated sequence of the sequences of two initial special nodes is assigned (Fig. 1a). Hereafter, for the breakpoint graph, an edge removal indicates the removal of only its internal part. *Sesqui-cut-and-paste* is the removal of an edge and joining in a new way with an edge with the same label of one of its free ends with a conventional free node non-incident to an edge with this label or with a special node of degree not exceeding 1 with the same label (which can be followed by a similar replacement of two special nodes). *Join* is inserting an edge (say with the label *a*) between free nodes, where each node is either conventional non-incident to an edge labeled *a* or special of degree not exceeding 1 with the same label (which can also be followed by the subsequent replacement of the special nodes if any). *Cut* is the removal of any edge.

In addition, only one *supplementary* operation on breakpoint graphs is allowed (it corresponds to the deletion operation on a structure): the *removal* of a special node (i.e., a block). Specifically, if this node *s* has the degree 2, it is removed and the edges incident to it are combined into one edge labeled as the neighbors of node *s* (Fig. 1b); if the node has degree 1, it is removed together with the edge incident to it (the conventional node is preserved); and if the node has degree 0 or has a loop, the isolated node and the loop are removed.

In [16, 23], we have reduced the problem of structure *a* transformation into structure *b* using the above six operations with allowed unequal gene content (without paralogs) to the problem of their breakpoint graph *a + b* transformation into the final form using these five operations. For equal gene contents, such transformation was proposed in [25]; for unequal gene contents without paralogs, this idea was implemented in [12].

### Statements of two problems

Hereafter, the structures can always have *unequal gene content* and *include paralogs*. The identification of paralogs (e.g., paralogs of a gene with the name *k*) means that they are given *unique* new names *k*.1, *k*.2, …. This form of paralog identification will be referred to as *numbering* of paralogs, and new names of the form *k.j* will be referred to as *full names* (of paralogs of gene *k*). The numbering makes it possible to establish a partial bijection between two sets of paralogs of gene *k* that belong to structures *a* and *b*, respectively. It is only partial since paralogs can disappear and emerge in the course of transformation (*a* to *b*) or evolution. If a gene has no paralogs, we can take that it has no index *j* or, better, assign it the same fixed index, e.g., 1.

It is important that the definitions of the common and special genes depend on the numbering of all paralogs of all genes, i.e., on the index *j*. Different paralog numberings in structures *a* and *b* can substantially change the breakpoint graph and its transformation to the final form.

At first, we define two problems to solve; the former is the *distance* problem. We are given two structures *a* and *b* with different gene content and paralogs. It is required to number paralogs of all genes in the structures to minimize the distance between the resulting structures without paralogs as well as to calculate this distance and to find the minimum sequence of operations.

The latter is the *reconstruction* problem. We are given a root and, generally speaking, non-binary tree *T*. Structures *a*_1_, …, *a*_*n*_ with different gene content and paralogs are defined in the tree leaves (their quantity is *n*). It is required to number all paralogs in the leaves and to identify mutually coherent numbered structures (in the inner nodes) with the minimum *total distance* calculated as the sum of distances for all edges of the tree, as well as to calculate the total distance. Only the names *k* present in the leaves are allowed in the inner nodes, and the upper limit *s*(*k*) of the index *j* is fixed for each *k* in these a priori unknown structures. Clearly, the appearance of new names in the inner nodes will not decrease the total distance. The distance on each edge is calculated as in the former problem. *Arrangement* is the assignment of a numbered structure to each node of the tree so that the leaves are assigned the initial predefined structures. Given the arrangement, the node and its structure are not distinguished. The minimum point of the specified function of the total distance in the latter problem is called the *minimum arrangement*, which is wanted; if there are several minimum points, we consider any one of them. Let F*(*A*) be the total distance at any arrangement *A*.

Section 2 presents an exact algorithm to solve the distance problem through its reduction to ILP. Section 3 presents an exact algorithm to solve the reconstruction problem by the same reduction *if* there is a minimum point (a minimum arrangement) for objective function *F′*, such that at the point, for any tree edge and for any circular chromosome at one of the edge ends, there is a gene from this chromosome present at the other end of the edge. This *condition* is applicable only to the problem of reconstruction and is marked by (*). Without this condition, our algorithm gives only an approximation *F′* to the minimum value F*; the difference between *F′* and F* is majorized.

The more general statement of the distance problem, which was considered, in particular, in [17, 23, 24], assigned each operation a weight, a strictly positive rational number, and the sequence transforming *a* into *b* with the minimum *total weight* of operations is sought. This generalization of the reconstruction problem is considered in [23, 24] on the basis of a direct algorithm and also can be reduced to ILP in a similar way as here. The latter more general consideration is omitted here for brevity. We have demonstrated that the problem of finding such total weight and the corresponding sequence of operations in this setup of the problem is reduced to the problem of breakpoint graph transformation to the final form if the weights of all standard operations are equal or obeyed some other constraints [16, 23].

The problem of *contigs* is to find the optimal concatenations of each given set of contigs providing their unequal gene content and identification of paralogs (see Section 4.1).

## Method and results

### Solution of the distance problem

#### Linear minimized function and its linear constraints

Below a reduction algorithm for the distance problem to integer linear programming (ILP) is described. We formulate the objective function *F*, variables and constraints of the ILP task, and also prove the key equality (1) in the Theorem 1.

Let *a* and *b* are given chromosome structures with unequal gene content and paralogs. Let us do arbitrarily numberings for gene paralogs as well as for genes without paralogs; the resulting numbered structures will be denoted as *a′* and *b′*. The numberings are called *initial*. We will deal only with numbered structures below. Let *adjacency* denote a pair of merged gene extremities that is a node of degree 2 in *a′* or *b′*.

Let us introduce Boolean variables *z*_*kij*_ to indicate whether genes *k.i* in *a′* and *k.j* in *b′* correspond to each other in terms of a partial bijection of paralogs in *a′* and *b′*; thus *z*_*kij*_ = 1 if *i* corresponds to *j*, otherwise *z*_*kij*_ = 0. Specifically, $\sum \limits_i{z}_{kij}\le 1$ for any fixed indexes *k* and *j*; and analogously for the sum over index *j*. Based on biological considerations, lower bounds can be set on this sum, e.g., $1\le \sum \limits_{i,j}{z}_{kij}$ for certain values of *k*.

A gene is called *common* if it becomes common after paralogs in *b′* are renumbered according to the *z*_*kij*_ values. Specifically, if *z*_*kij*_ = 1, the gene *k.j* in *b′* is renamed to *k.i* and becomes synonymous to *k.i* in *a′*, after which the genes out of the *z*-bijection are arbitrarily numbered to keep the structures numbered. Similarly, a gene is called *special* if it becomes special after renumbering. The structures resulting from such renumbering in *b′* will be referred to as *a′*(*z*) and *b′*(*z*). A circular chromosome composed of only special genes will be called *special*. Circular chromosome will be referred to as *1-circular* if it composed of a single gene; otherwise it is *m-circular*. For each circular chromosome *d* in *a′*, let us define $o\left(d,a\right)=\left(\sum \limits_{k.i\in d,k.j\in {b}^{\prime }}{z}_{kij}\right)/{n}_d$ where *n*_*d*_ is the quantity of genes in *d*. For a linear chromosome *d*, we set *o*(*d*) = 1; 0 ≤ *o*(*d*) ≤ 1. It holds that *d* is special if and only if *o*(*d*,*a*) = 0. The value of *o*(*d*,*a*) indicates the proportion of genes in *d* that are in *z*-bijection with genes in *b′*. The proportion *o*(*d*,*b*) for a chromosome *d* in *b′* is defined similarly. References to *a* or *b* are usually omitted.

Let us equalize the gene contents in *a′*(*z*) and *b′*(*z*) just by adding to *a′*(*z*) special *b′*(*z*)-genes except the genes from special *b′*(*z*)-chromosomes; a similar addition is made to *b′*(*z*). All added genes are combined into circular chromosomes, some from *a′*(*z*) and some from *b′*(*z*). The resulting chromosomes as well as their genes and gene adjacencies will be referred to as *new*. New adjacencies are defined by a new variable *t*, which is formally described below. Thus obtained structures referred to as *a* ^*−*^(*z*,*t*) and *b* ^*−*^(*z*,*t*) released from special chromosomes (if any) are denoted as *a″*(*z*,*t*) and *b″*(*z*,*t*). Let us introduce the breakpoint graph

$$ {G}^{\prime}\left(z,t\right)={a}^{\prime \prime}\left(z,t\right)+{b}^{\prime \prime}\left(z,t\right) $$

It is proved as in [12] that the distance between *a* ^*−*^(*z*,*t*) and *b* ^*−*^(*z*,*t*) equals Φ(*z*, *t*) for any *z* and *t*. It follows that, for a fixed *z*, the minimum by *t* distance between *a* ^*−*^(*z*,*t*) and *b* ^*−*^(*z*,*t*) equals min_*t*_Φ(*z*, *t*); for any *z*, *t*_0_ = *t*_0_(*z*) defines the value of *t* corresponding to this minimum. Here

$$ \Phi \left(z,t\right)=\left({C}_0+n+{s}_a+{s}_b\right)-{\mathrm{C}}_1-0.5{C}_2, $$

where *C*_0_ is the total number of special chromosomes in *a′*(*z*) and *b′*(*z*), *C*_1_ is the number of cycles in *G′*, *C*_2_ is the number of even paths in *G′*, *n* is the number of common genes in *a′*(*z*) and *b′*(*z*) counted once, and *s*_*a*_, *s*_*b*_ are the quantities of new genes in *a* ^*−*^(*z*,*t*) and *b* ^*−*^(*z*,*t*). *Even* (odd) path is a path of even (odd) length. Notice that natural constraints are imposed on *z* and *t* in the definition of Φ. Following [12], it is easy to verify that the distance between *a* ^*−*^(*z,t*_0_) and *b* ^*−*^(*z,t*_0_) equals the distance between *a′*(*z*) and *b′*(*z*) for any *z*. There is no *z* variable in [12] since paralogs are not considered there; the *t* variable is not used either. Thus, solving the distance problem requires finding min_*z*_min_*t*_Φ(*z*, *t*). By definition, a new adjacency corresponds to the *new edge* in *G′*(*z*); the remaining edges in *G′* are called *old*.

Now let us define the variable *t* which describes new adjacencies. For each pair *s* = (*g*,*g′*) of different gene extremities in *a′*, we define a Boolean variable *t*_*bs*_ to indicate whether *g* and *g′* form a new adjacency in *b″*(*z,t*). Specifically, ${t}_{bs}\le 1-\sum \limits_j{z}_{kij}$, *t*_*bs*_ ≤ *n*_*g*_ ⋅ *o*(*d*_*g*_), $\sum \limits_{g^{\prime }}{t}_{bg{g}^{\prime }}\le 1$, and $\sum \limits_{g^{\prime }}{t}_{bg{g}^{\prime }}\ge o\left({d}_g\right)-\sum \limits_j{z}_{kij}$, where *k*.*i* is a gene with the extremity *g*, *d*_*g*_ is the chromosome containing *k.i*, *n*_*g*_ is quantity of genes in *d*_*g*_. Similar variable *t*_*as*_ and constraints are defined for extremities in *b′*. Often we will omit the indexes *a* and *b* near *t*.

Items 1–3 below describe the summands of the function Φ by means of equivalent ILP formulation (of minimization). To this end, let us sequentially describe the summands *C*_1_, *C*_2_, and *C*_0_ + *n* + *s*_*a*_ + *s*_*b*_ in Φ. Thus, the objective function will be equal to

$$ F=\left(\sum \limits_d{n}_d+\sum \limits_d\left(1-{n}_d\right){o}_d-\sum \limits_{k,i,j}{z}_{kij}\right)-\sum \limits_s{p}_s-0.5\left(\sum \limits_g{r}_g-\sum \limits_g{l}_g\right) $$

where *d* runs over all chromosomes in *a′* or *b′* and *n*_*d*_ is the quantity of genes in chromosome *d*. The summand $\sum \limits_d{n}_d$ is a constant and has no effect on the minimum value. The variables *o*_*d*_, *p*_*s*_, *r*_*p*_, *l*_*p*_ and their linear constraints will be defined in items 1–3 below. The critical point is the equality

1 $$ \underset{z,t}{\min}\varPhi \left(z,t\right)=\min \kern0.40em F\left(o,z,p,r,l\right). $$

1. Here we use the counting cycles idea from [5]. Let us describe the quantity *C*_1_ of cycles in the breakpoint graph *G′*. Let us do numbering of all adjacencies (*g*,*g′*) in *a′* and *b′* starting from one; and *m*_*s*_ is the number of an adjacency *s*. Let us for each *s* introduce an integer (non-Boolean) variable *u*_*s*_ with the constraint 0 ≤ *u*_*s*_ ≤ *m*_*s*_. We require that *u*_*s*_ = 0 for all adjacencies *s* in *a′* from special chromosomes *d* in *a′*(*z*); with regard to other constraints, it is expressed as the inequality ${u}_s\le {m}_s\sum \limits_{k.i\in d}\sum \limits_j{z}_{kij}$ for any circular chromosome *d*. And symmetrically for adjacencies in *b′*.

Two extremities of two genes are defined to be of the *same type* if both of them are either 5′-ends or 3′-ends and belong to paralogs in different structures. We require that *u*_*s*_ = 0 for any adjacency *s* in *a′* such that one of its extremities belongs to a common gene and is a boundary of a path in *G′*. Specifically, let *g* be an extremity of gene *k*. *i* ∈ *a* ^′^ adjacent to any extremity in *s*. For each gene *k*.*j* in *b′* with an extremity of the same type as *g* that is a boundary of a path in *b′*, the constraint *u*_*s*_ ≤ *m*_*s*_(1 − *z*_*kij*_) is imposed. The constraints are symmetrical for *b′*.

Further, we require that *u*_*s*_ = 0 for any adjacency *s* in *a′* such that one of its extremities belongs to a special *a*-gene and is not a boundary of a path through the end of a terminal new edge of a path in *G′*. Specifically, for each extremity *g*_1_ in *a′* that is a boundary of a path in *a′*, we impose that *u*_*s*_ ≤ *m*_*s*_(1 − *t*_*g*1*g*_) where *s* includes *g*. The constraints are symmetrical for *b′*.

We require that *u*_*s*_ is constant at all edges in a cycle or path in *G′*. Specifically, for each pair of adjacencies *s*1 = (*g*,*g*_1_) and *s*2 = (*g′*,*g*_2_) in *a′* and *b′*, respectively, with *g* and *g′* being of the same type, we impose

$$ {u}_{s1}\le {u}_{s2}+{m}_{s1}\left(1-{z}_{kj{j}^{\prime }}\right),{u}_{s2}\le {u}_{s1}+{m}_{s2}\left(1-{z}_{kj{j}^{\prime }}\right) $$

where *k*.*j* and *k*.*j′* are genes with the extremities *g* and *g′*. These two constraints ensure that *u*_*s*1_ = *u*_*s*2_ for two neighboring edges *s*1 and *s*2 in *G′* that are both old edges. For each pair of different adjacencies *s*1 = (*g*_1_,*g*_2_) and *s*2 = (*g*_3_,*g*_4_) of extremities both in *a′* or *b′*, we impose that *u*_*s*1_ ≤ *u*_*s*2_ + *m*_*s*1_(1 − *t*_*g*2*g*3_), *u*_*s*2_ ≤ *u*_*s*1_ + *m*_*s*2_(1 − *t*_*g*2*g*3_). These constraints ensure that *u*_*s*1_ = *u*_*s*2_ for two edges in *G′* that are both old edges and spaced by exactly one new edge.

For each adjacency *s*, we define the Boolean variable *p*_*s*_ to indicate whether *u*_*s*_ is equal to its upper bound *m*_*s*_ at the minimum point of the function *F*. Specifically, *p*_*s*_∙*m*_*s*_ ≤ *u*_*s*_. Indeed, if *u*_*s*_ *< m*_*s*_, then *p*_*s*_ = 0. Otherwise, *p*_*s*_ can take any of two values, but since variables *p*_*s*_ are summands of *F* with negative coefficients, we have *p*_*s*_ = 1.

Since *u*_*s*_ has a constant value on all edges in a cycle and all upper bounds are unequal, there is exactly one edge at the minimum point whose *u*_*s*_ equals its upper bound. Indeed, exactly one of *p*_*s*_ equals 1 in a cycle at the minimum point. In a path, the constraints imply that *u*_*s*_ = 0 so that neither of them can reach the maximum; hence, *p*_*s*_ = 0 in a path. Considering that any cycle contains at least one old edge, the quantity of variables *u*_*s*_ that reaches its maximum is equal to the quantity of cycles, thus ${C}_1=\sum \limits_s{p}_s$ at the minimum point of *F*.

2) Let us describe the quantity *C*_2_ of *even* paths in the graph *G′*. Let us introduce three-valued (0, 1 or −1) integer variables *r*_*ag*1_ and *r*_*bg*2_ for any gene extremity *g*_1_ and *g*_2_ in *a′* and *b′* such that, at the minimum point of *F*, the *sum* of the variables (if *g*_1_ and *g*_2_ are in *z*-bijection and have the same type, *r*_*bg2*_ is omitted) by the nodes of a path or a cycle in *G′* equals 1 if it is an even path; otherwise it equals 0. At the minimum point of *F*, it follows from the constraint that the values of *r* at adjacent nodes in *G′* are not equal to 1 and 1 or 0 and 1. Specifically, for each adjacency (*g*_1_,*g*_2_) in *a′* or *b′*, we impose that *r*_*ag*1_ + *r*_*ag*2_ ≤ 0 or *r*_*bg*1_ + *r*_*bg*2_ ≤ 0, respectively. For each pair of different extremities *g*_1_ and *g*_2_ from *a′* which do not form an adjacency, we impose that *r*_*ag*1_ + *r*_*ag*2_ ≤ 2(1 − *t*_*ag*1*g*2_). Similar constraints are imposed for *b′*. For each pair (*g*,*g′*) of extremities of the same type from *a′* and *b′*, respectively, we impose that $-2\left(1-{z}_{kj{j}^{\prime }}\right)\le {r}_g-{r}_{g^{\prime }}\le 2\left(1-{z}_{kj{j}^{\prime }}\right)$, where *k*.*j* and *k*.*j′* are genes with extremities *g* and *g′*. These constraints ensure that *r*_*g*_ + *r*_*g*'_ ≤ 0 if (*g*,*g′*) is an edge in *G′*; also if *g* and *g′* are in *z*-bijection, then *r*_*ag*_ = *r*_*bg′*_. Considering that the variables *r*_*g*_ are summands of *F* with some negative coefficients, they equal 1 at the minimum point at isolated nodes in *G′*. The lengths of cycles in *G′* are even, and the values of *r*_*g*_ in their nodes either alternate between 1 and −1 or constantly equal 0. Therefore, the above sum along a cycle equals 0. The *r*_*g*_ values alternate on nonzero even paths being equal to 1 at the path boundaries; accordingly, the sum along an even path equals 1. On an odd path, such alternation can be interrupted by zero values, but again the sum along its nodes equals 0. Hence, it follows that the sum indicates each even path. For a special chromosome *d*, $\sum \limits_{g\in d}{r}_g=0$ at the point of minimum of *F* since this sum is clearly not greater than 0.

Let us define the sum described in the beginning of item 2. For each extremity *g* of a gene in *a′*, we define an integer variable *l*_*g*_, which equals *r*_*ag*_ if *g* is an extremity of a common gene, or equals 0 otherwise. This is provided by the constraints $-\sum \limits_j{z}_{kij}\le {l}_g\le \sum \limits_j{z}_{kij}$, ${l}_g\le {r}_{ag}+2\left(1-\sum \limits_j{z}_{kij}\right)$, ${r}_{ag}\le {l}_g+2\left(1-\sum \limits_j{z}_{kij}\right)$, where *k.i* is a gene with extremity *g*. Thus, the node *g* in *G′*, an extremity of a common gene, corresponds to three variables *r*_*ag*_, *r*_*bg*_, and *l*_*g*_, which take equal values. This allows us to cancel the summands *r*_*ag*_ and –*l*_*g*_ when summing up all *r*_*ag*_, *r*_*bg*_, and –*l*_*g*_. The node *g*, an extremity of a special gene in *a′*(*z*), corresponds to two variables *r*_*ag*_ and *l*_*ag*_, the latter equals 0. The node *g*, an extremity of a special gene in *b′*(*z*), corresponds to one variable *r*_*bg*_. Therefore, ${C}_2=\sum \limits_g{r}_g-\sum \limits_g{l}_g$ in a minimum point of *F*.

3) Let us describe the summand *C*_0_ + *n* + *s*_*a*_ + *s*_*b*_. For each chromosome *d* in *a′* or *b′*, we define a Boolean variable *o*_*d*_ to indicate whether this chromosome is special *m*-circular at the minimum point of *F*. Specifically, if *d* is *m*-circular then *o*_*d*_ ≤ 1 − *o*(*d*); if *d* is a 1-circular or a linear chromosome, then *o*_*d*_ = 0. Indeed, *o*_*d*_ = 0 follows from the above constraint if *d* is not special or is special and 1-circular. For a special *m*-circular chromosome *o*_*d*_ = 1 at the minimum point of *F* considering that variables *o*_*d*_ are summands of *F* with negative coefficients.

Let us show that in a minimum point of *F* we have

$$ {C}_0+n+{s}_a+{s}_b=\sum \limits_d{n}_d+\sum \limits_d\left(1-{n}_d\right){o}_d-\sum \limits_{k,i,j}{z}_{kij}, $$

where *d* runs over all chromosomes in the first sum and over all *m*-circular chromosomes in the second sum, and *n*_*d*_ is the quantity of genes in *d*. The number *n* is equal to the sum of all *z*_*kij*_ values, while the numbers *s*_*a*_ and *s*_*b*_ are equal by the definition as follows: *s*_*a*_ = *n*_*b*_ – *n* and s_*b*_ = *n*_*a*_ – *n*, where *n*_*a*_ and *n*_*b*_ are quantities of genes in structures *a′*(*z*) and *b′*(*z*), respectively, not in special chromosomes. Thus, *n* + *s*_*a*_ + *s*_*b*_ = *n*_*a*_ + *n*_*b*_ – *n.* Considering that ${C}_0=\sum \limits_d{o}_d+U$, $n=\sum \limits_{kij}{z}_{kij}$, and ${n}_a+{n}_b=\sum \limits_d{n}_d\left(1-{o}_d\right)-U$, where *U* is the quantity of 1-circular chromosomes, the desired equality is readily derived from the previous equality.

##### Theorem 1

For given *a* and *b*, the minimum paralog numbering and minimum value of the distance are defined by the minimum point of *F*.

##### Proof

Let the function *F* reaches the minimum at the point *x*_0_. It follows from items 1–3 that the function Φ(*z*, *t*) calculated at the point *y*_0_ = (*z*_0_,*t*_0_), which is a part of *x*_0_ coordinates, equals *F*(*x*_0_). Such *y*_0_ is the minimum for Φ(*z*, *t*). Indeed, if there is (*z*,*t*), for which the value of Φ(*z*, *t*) is strictly lower, then (*z*,*t*) can be extended to the point where *F* is equal to Φ, which is impossible. The extension is as follow. The point (*z*,*t*) together with given *a′* and *b′* uniquely define *G′*; *p*, *r*, *l* are defined by *G′*; and *o*_*d*_ is defined by *a′*(*z*) and *b′*(*z*). □.

Clearly, the number of variables and constraints in it quadratically depends on the data size of the initial problem.

##### Note 1

After solving the ILP task, one can use (as in [16]) the obtained *z* and the structures *a′*(*z*) and *b′*(*z*) to find the minimum sequence of operations transforming *a′*(*z*) into *b′*(*z*).

### Examples for the distance problem based on synthetic data

#### Example 1

Let the structure *a* include three circular chromosomes with unidirectional genes: (1, 3); (1, 2, 2); (3, 5, 2, 4) and the structure *b* also include three circular chromosomes: (4, 2); (1, 2, 1); (4, 5, 5, 3) with unidirectional genes. Let us introduce the initial numbering; for *a′*, it is (1.1, 3.1); (1.2, 2.1, 2.2); (3.2, 5.1, 2.3, 4.1); for *b′*, it is (4.1, 2.1); (1.1, 2.2, 1.2); (4.2, 5.1, 5.2, 3.1). The ILP program of the Pulp python package returned the following solution: the number of operations transforming *a′* into *b′* equals 4. At the minimum point, the paralogs in *b′* are renumbered as follows: 1.1 to 1.2, 1.2 to 1.1, 2.1 to 2.3, 2.2 to 2.1, 3.1 to 3.2, 5.1 to 5.2, 5.2 to 5.1. The program execution time was about 1.5 h.

#### Example 2

We are given two structures with the following arrangement of genes on the chromosomes; *a*: (1, 2, −3, 4, 5, 6), (3), [10], [−7, 8, 9] and *b*: (1), (2), (9), (4, 6, −3, 5), [8], [−7, 10, 3]. Here minus sign indicates the complementary strand, while round and square brackets indicate circular and linear chromosomes, respectively. The initial numberings are as follows; *a′*, the gene 3 is 3.1 and 3.2 in the large and small cycles, respectively; *b′*, the gene 3 is 3.1 and 3.2 in the path and cycle, respectively. The ILP program of the Pulp python package returned the following solution: the number of operations transforming *a′* into *b′* equals 7. At the minimum point the paralogs in *b′* are renumbered as follows: 3.1 to 3.2, 3.2 to 3.1. The program execution time was about 3 h.

## Solution of the reconstruction problem

Below a reduction of the algorithm for the reconstruction problem to integer linear programming (ILP) is described. We formulate the objective function *F′*, variables and constraints of the ILP task, while the Theorem 2 proves that ILP can solve the problem. Let *T* be a fixed rooted possibly non-binary tree. Recall that *leaf* edge link to a tree leaf and *inner* edge means a non-leaf tree edge. *T*-Edge and *G″*-edge emphasize that this edge belongs to *T* and *G″*, respectively, but not to any structure. The structure in a node *x* is usually denoted by *x*; in this sense we do not distinguish a node and its structure.

### Linear minimized function and its linear constraints

The argumentation is largely the same as in the distance problem fully described in Section 2 above, and it will not be reproduced in detail here. The specialties distinguishing the solution of the reconstruction problem from that of the distance one will be emphasized. Hereafter, *a* and *b* are nodes and, at the same time, structures in the beginning and end of a *T*-edge, respectively; an edge is often designated as *e* = (*a*,*b*). Let us fix the initial paralog numberings in all given structures assigned to the leaves; they are called *initial*. For a leaf *b*, the given initially numbered structure is designated as *b′*, while any numbered structure is designated as *u′*, *a′*, and likewise. Let *M* denote a set of all full names *k.i*, where 1 ≤ *i* ≤ *s*(*k*). Recall that circular chromosomes composed solely of special genes are called *special*.

We define the variable *z*_*ukij*_ for each leaf *u* and each gene *k*.*i* from *u′* and *k*.*j* from *M*; it equals 1 if *k*.*i* is renamed to *k*.*j*; otherwise *z*_*ukij*_ = 0. The existence and uniqueness of *k*.*j* is ensured by the following constraints:

$$ \mathrm{for}\  \mathrm{fixed}\ k\ \mathrm{and}\ i,\sum \limits_j{z}_{ukij}=1;\mathrm{for}\  \mathrm{fixed}\ k\ \mathrm{and}\ j,\sum \limits_i{z}_{ukij}\le 1. $$

The index *u* is usually omitted.

We define the variable *y*_*vk.i*_ for each inner node *v* and each gene *k.i* from *M*; it equals 1 if *k.i* is missing from *v*; otherwise it equals 0. For each inner node *v* and each pair (*g*,*g′*) of different extremities from *M*, we define the variable *x*_*vgg′*_; it equals 1 if *g* and *g′* are present and merged in the node *v*; otherwise it equals 0. The variables *x*_*vgg′*_ are not specified in leaves since their values are fixed there. Specifically, $\sum \limits_{g^{\prime}\ne g}{x}_{vg{g}^{\prime }}\le 1-{y}_{vk.i}$ implies that any extremity *g* of any gene *k*. *i* ∈ *M* missing in *v* is not merged, where *g′* runs over all extremities from *M*; and the constraint implies that $\sum \limits_{g^{\prime }}{x}_{vg{g}^{\prime }}\le 1$ for any fixed *v* and *g*. The index *v* is usually omitted.

In order to avoid degenerate scenarios with empty ancestral structures, we lay the condition that if a gene is absent from an inner node *v*, it is absent from at least a half of its direct descendants. Specifically, the following constraint is imposed on each name *k.j* from *M*:

$$ {y}_{vk.j}\le 1.5-\frac{1}{n_v}\left[\sum \limits_{v^{\prime }}\left(1-{y}_{v^{\prime }k.j}\right)+\sum \limits_{v^{\prime }}\sum \limits_i{z}_{v^{\prime } kij}\right], $$

where *n*_*v*_ is the total number of direct descendants *v′* of *v*; in the first and second sums, *v′* runs over the inner nodes and leaves, respectively. This constraint can be simplified for a binary tree:

$$ {y}_{vk.j}\le w\left({v}^{\prime}\right)+w\left({v}^{{\prime\prime}}\right), $$

where *v′* and *v″* are direct descendants of the node *v,* and $w\left({v}^{\alpha}\right)={y}_{v^{\alpha }k.j}$ if *v* ^*α*^ is not a leaf or $w\left({v}^{\alpha}\right)=1-\sum \limits_i{z}_{v^{\alpha } kij}$ otherwise.

As in Section 2 we equalize the gene contents in *a′*(*z*) and *b′*(*z*) where the variable *z* defines identical bijections for inner edges. But now we add to *a′*(*z*) all special *b′*(*z*) genes; respectively, to *b′*(*z*) all special *a′*(*z*) genes; we denote obtained structures *a* ^+^(*z*,*t*) and *b* ^+^(*z*,*t*). Thus, special chromosomes are not removed. Therefore the breakpoint graph *G″* of *a* ^+^(*z*,*t*) and *b* ^+^(*z*,*t*) may be different from the graph *G′* defined in Section 2.

For each edge *e* = (*a*,*b*) and each pair *s* = (*g*,*g′*) of different gene extremities from *M* we define the Boolean variable *t*_*ebs*_ to make sure that if *t*_*ebs*_ = 1, then *g* and *g′* form a new adjacency in *b* ^+^(*z*,*t*)*.* Similar variable *t*_*eas*_ is introduced for *a*, but if *b* is a leaf, *t*_*eas*_ is defined only for the extremities present in *b′*. The index *e* can be omitted. Let *k*.*j* be a gene with extremity *g*. For a leaf edge *e*, the constraints are as follows:

$$ {\displaystyle \begin{array}{l}{t}_{ebs}\le 1-{y}_{ak.j},{t}_{ebs}\le 1-\sum \limits_i{z}_{bkij},\sum \limits_{g1\in M}{t}_{ebgg1}\le 1,\sum \limits_{g1\in {b}^{\prime }}{t}_{eagg1}\le 1,\sum \limits_{g1\in M}{t}_{ebgg1}\ge 1-{y}_{ak.j}-\sum \limits_i{z}_{bkij};\\ {}{t}_{eas}\le 1+{y}_{ak.\alpha }-{z}_{bkj\alpha},\sum \limits_{g1\in {b}^{\prime }}{t}_{eagg1}\ge {y}_{ak.\alpha }+{z}_{bkj\alpha}-1.\end{array}} $$

Actually, the last two constraints assume the systems of inequalities for each value of *α*, such that 1 ≤ *α* ≤ *s*(*k*).

For an inner edge *e*, we impose that:

$$ {t}_{ebs}\le 1-{y}_{ak.j},{t}_{ebs}\le {y}_{bk.j},\sum \limits_{g1\in M}{t}_{ebgg1}\le 1,\sum \limits_{g1\in M}{t}_{bgg1}\ge {y}_{bk.j}-{y}_{ak.j}. $$

Similar constraints are imposed for *t*_*eas*_.

For any leaf edge *e* ∈ *T*, let |*M*| be the quantity of elements in *M*, and *c*_*e*_ be |*M*| plus the quantity of genes in *b*. The objective *function F′* (for the task of minimization) equals the sum of two expressions. The first one is the sum

$$ \left({c}_e-\sum \limits_{k.i\in M}{y}_{ak.i}-\sum \limits_{k.j\in M}{f}_{k.j}\right)-\sum \limits_s{p}_s-0.5\left(\sum \limits_g{r}_g-\sum \limits_g{l}_g\right) $$

calculated over all leaf *T*-edges *e*. The second one is the sum

$$ \left(2\cdot |M|-\sum \limits_{k.i}{y}_{ak.i}-\sum \limits_{k.i}{y}_{bk.i}-\sum \limits_{k,j}{f}_{k.j}\right)-\sum \limits_s{p}_s-0.5\left(\sum \limits_g{r}_g-\sum \limits_g{l}_g\right) $$

calculated over all inner *T*-edges *e*. The variables except *y* and corresponding constrains are defined in the following items 1–3. They correspond to items 1–3 in Section 2, which described the algorithm of reduction for the distance problem.

1. Let *e* = (*a*,*b*) be a *T*-edge and *G″*(*e*) = *a* ^+^(*z*,*t*) + *b* ^+^(*z*,*t*). Let us define the variables *u*_*es*_ and *p*_*es*_ as well as the constraints ensuring that the number *C*_1_ *′* of cycles in the graph *G″*(*e*) at the minimum point of *F′* equals $\sum \limits_s{p}_{es}$. Specifically, for each pair *s* = (*g*,*g′*) of different extremities from *M* for an inner edge *e* = (*a*,*b*), we define the integer non-negative variables *u*_*eas*_ and *u*_*ebs*_ and Boolean variables *p*_*eas*_ and *p*_*ebs*_. For a leaf edge *e* and its *b′*, we define the integer non-negative variable *u*_*ebs*_ and Boolean variable *p*_*ebs*_, where *s* is any adjacency in *b′*. Both variables *u*_*eas*_ and *u*_*ebs*_ obey *u*_*s*_ ≤ *m*_*s*_. Here, *m*_*s*_ is the *number* of the mentioned pair *s*, where *s* runs over all pairs where the variables *u*_*eas*_ and *u*_*ebs*_ are defined for any fixed *e* ∈ *T*. For Boolean variable *p*_*es*_, we impose that *p*_*es*_∙*m*_*s*_ ≤ *u*_*s*_.

Let *e* = (*a*,*b*) be a leaf edge. We impose that *u*_*as*_ ≤ *m*_*as*_ ⋅ *x*_*as*_ ensuring that *u*_*as*_ = 0 for any pair *s* of non-merged extremities from *M*. For *a*, let *s* include *g* which is an extremity of a gene *k*.*j* from *M*. Each variable *u*_*as*_ and each extremity of a gene *k*. *j* ^′^ ∈ *b* ^′^ of the same type as *g* and a boundary of a path in *b′* are imposed that ${u}_{as}\le {m}_{as}\left(1-{z}_{k{j}^{\prime }j}\right)$. These constraints ensure that *u*_*s*_ = 0 if the extremity *g* belongs to a common gene of *a′*(*z*) and *b′*(*z*), and in *G″*(*e*) we have: *g* is a boundary of a path and, at the same time, is an extremity of an *G″*-edge marked *a*. For *b*, let an adjacency *s* ∈ *b* ^′^ and includes *g* ∈ *k*. *j*. Each variable *u*_*bs*_ and each *i* (1 ≤ *i* ≤ *s*(*k*)) are imposed that ${u}_{bs}\le {m}_{bs}\left(1-{z}_{kji}+\sum \limits_{g1\in M}{x}_{a{g}^{\prime }g1}\right)$, where *g′* is the extremity of a gene *k*. *i* ∈ *M* of the same type as *g*. These constraints ensure that *u*_*s*_ = 0 if *g* belongs to a common gene of *a′*(*z*) and *b′*(*z*), and in *G″*(*e*) we have: *g* is a boundary of a path and, at the same time, an extremity of a *G″*-edge marked *b*. Each extremity *g*_1_ from *M* is imposed that ${u}_{as}\le {m}_{as}\left(1-{t}_{bg1g}+\sum \limits_{g2}{x}_{ag1g2}\right)$, which ensures that *u*_*s*_ = 0 if *g* ∈ *s*, *g* belongs to a special gene in *a′*(*z*) and *g* in *G″*(*e*) is not a boundary of a path but the end of a terminal new *G″*-edge of the path. Each extremity *g*_1_ in *b′* that is a boundary of a path in *b′* is imposed the constraint *u*_*bs*_ ≤ *m*_*as*_(1 − *t*_*ag*1*g*_), ensuring that *u*_*bs*_ = 0 if the extremity *g* ∈ *b* ^′^, *g* belongs to a special gene in *b′*(*z*) and *g* in *G″*(*e*) is not a boundary of a path but the end of a terminal new *G″*-edge of the path.

Recall that now we consider a leaf edge *e* = (*a*,*b*). Each pair (*s*1,*s*2), where *s*1 = (*g*,*g*_1_) is a pair of extremities from *M* and *s*2 = (*g′*,*g*_2_) is an adjacency from *b′* where *g* and *g′* are of the same type and belongs to paralogs *k*.*j* and *k*.*j′*, is imposed the constraints:

$$ {u}_{as1}\le {u}_{bs2}+{m}_{as1}\left(1-{z}_{k{j}^{\prime }j}\right),{u}_{bs2}\le {u}_{as1}+{m}_{bs2}\left(2-{z}_{k{j}^{\prime }j}-{x}_{agg1}\right). $$

It follows that *u*_*s*1_ = *u*_*s*2_ for neighboring old *G″*-edges *s*1 and *s*2 of *G″*(*e*). Each pair (*s*1,*s*2), where *s*1 = (*g*_1_,*g*_2_) and *s*2 = (*g*_3_,*g*_4_) are pairs of extremities from *M*, is imposed that

$$ {u}_{as1}\le {u}_{as2}+{m}_{as1}\left(2-{t}_{bg2g3}-{x}_{ag3g4}\right),{u}_{as2}\le {u}_{as1}+{m}_{as2}\left(2-{t}_{bg2g3}-{x}_{ag1g2}\right), $$

all *g*_1_, *g*_2_, *g*_3_, *g*_4_ are pairwise different. These constraints ensure that *u*_*s*1_ = *u*_*s*2_ for two old *G″*-edges (marked *a*) of *G″*(*e*) spaced by exactly one new *G″*-edge. Each pair (*s*1,*s*2) where *s*1 = (*g*_1_,*g*_2_) and *s*2 = (*g*_3_,*g*_4_) are different adjacencies from *b′* are imposed that

$$ {u}_{s1}\le {u}_{s2}+{m}_{s1}\left(1-{t}_{ag2g3}\right),{u}_{s2}\le {u}_{s1}+{m}_{s2}\left(1-{t}_{ag2g3}\right). $$

These constraints ensure that *u*_*s*1_ = *u*_*s*2_ for two old *G″*-edges (marked *b*) of the graph *G″*(*e*) spaced by exactly one new *G″*-edge.

For an inner edge *e* = (*a*,*b*), let us impose that *u*_*as*_ ≤ *m*_*as*_ *x*_*as*_, *u*_*bs*_ ≤ *m*_*bs*_ *x*_*bs*_, ensuring that *u*_*as*_ *=* 0 or *u*_*bs*_ = 0 for non-merged *s* = (*g*,*g′*). Each variable *u*_*as*_ is imposed that ${u}_{as}\le {m}_{as}\left({y}_{bk.j}+\sum \limits_{g1}{x}_{bgg1}\right)$ where *s* includes *g* ∈ *k*. *j*. Similar constraints are imposed for *u*_*bs*_. It ensures that *u*_*s*_ = 0 if *g* belongs to a common gene and is a boundary of a path in *G″*(*e*). The equality *u*_*s*_ = 0 (for *u*_*as*_ and *u*_*bs*_) in the case when the extremity *g* belongs to a special gene (in *a′*(*z*) or *b′*(*z*)) and a boundary edge of a path (in *G″*(*e*)) is provided in the same manner as for *u*_*as*_ on a leaf edge. Each pair (*s*1,*s*2), where *s*1 = (*g*,*g*_1_) and *s*2 = (*g*,*g*_2_) are different pairs of extremities from *M*, is imposed that

$$ {u}_{as1}\le {u}_{bs2}+{m}_{s1}\left(1-{x}_{bgg2}\right),{u}_{bs2}\le {u}_{as1}+{m}_{s2}\left(1-{x}_{agg1}\right). $$

These constraints ensure that *u*_*s*1_ = *u*_*s*2_ for old neighboring *G″*-edges *s*1 and *s*2 in *G″*(*e*). Each pair (*s*1,*s*2), where *s*1 = (*g*_1_,*g*_2_) and *s*2 = (*g*_3_,*g*_4_) are pairs of extremities from *M*, is imposed that

$$ {\displaystyle \begin{array}{l}{u}_{as1}\le {u}_{as2}+{m}_{as1}\left(2-{t}_{bg2g3}-{x}_{ag3g4}\right),{u}_{as2}\le {u}_{as1}+{m}_{as2}\left(2-{t}_{bg2g3}-{x}_{ag1g2}\right),\\ {}{u}_{bs1}\le {u}_{bs2}+{m}_{bs1}\left(2-{t}_{ag2g3}-{x}_{bg3g4}\right),{u}_{bs2}\le {u}_{bs1}+{m}_{bs2}\left(2-{t}_{ag2g3}-{x}_{bg1g2}\right)\end{array}} $$

(all *g*_1_, *g*_2_, *g*_3_, *g*_4_ are pairwise different). These constraints ensure that *u*_*s*1_ = *u*_*s*2_ for two old *G″*-edges of the graph *G″*(*e*) spaced by exactly one new *G″*-edge.

The statement that ${C_1}^{\prime }=\sum \limits_s{p}_s$ at the minimum point, for any *e* ∈ *T*, is proved in the same way as in Section 2.

2) Let us define the variables and constraints ensuring that the quantity *C*_2_ *′* of even paths in *G″*(*e*) on an edge *e* = (*a*,*b*) at the minimum point of *F′* equals $\sum \limits_g{r}_g-\sum \limits_g{l}_g$. Let us define for each extremity *g* from *M* an integer variable *r*_*eag*_ that runs over the values 0, +1, −1. And similarly for *b* if *b* is inner; otherwise only for each extremity *g* in *b′.*

The constraint −2(1 − *y*_*ak*. *i*_) ≤ *r*_*eag*_ ≤ 2(1 − *y*_*ak*. *i*_) implies that *r*_*eag*_ = 0 for any extremity *g* of any gene *k*. *i* ∈ *M* missing in *v*. And similarly for *b* if *b* is inner.

Each pair of different extremities *g*_1_ and *g*_2_ from *M* is imposed that *r*_*eag*1_ + *r*_*eag*2_ ≤ 2(1 − *x*_*ag*1*g*2_), ensuring that *r*_*eag*1_ + *r*_*eag*2_ ≤ 0 if these extremities are merged. For an inner edge *e*, similar constraints are imposed with the index *a* replaced by *b*; otherwise, they are imposed only for each adjacency (*g*_1_,*g*_2_) from *b′* with zero in the right part. It is also imposed that *r*_*eag*1_ + *r*_*eag*2_ ≤ 2(1 − *t*_*ebg*1*g*2_), ensuring that *r*_*eag*1_ + *r*_*eag*2_ ≤ 0 if *g*_1_ and *g*_2_ form a new adjacency. For an inner edge *e*, similar constraints are imposed with the index *a* replaced by *b* and vice versa; otherwise this constraint is imposed only for pairs (*g*_1_,*g*_2_) of extremities from *b′* that do not form an adjacency. For a leaf edge *e*, each pair (*g*,*g′*), where extremities *g* (of *k*.*j*) and *g′* (of *k*.*j′)* are of the same type, *g* is from *M*, and *g′* is from *b′*, the constraint is imposed that

$$ -2\left(1-{z}_{bk{j}^{\prime }j}+{y}_{ak.j}\right)\le {r}_{eag}-{r}_{eb{g}^{\prime }}\le 2\left(1-{z}_{bk{j}^{\prime }j}+{y}_{ak.j}\right), $$

ensuring that ${r}_{ebg}={r}_{ea{g}^{\prime }}$ for *z*-bijection extremities *g* and *g′* of the same type if *g* is present in *a*. For an inner edge *e* and each extremity *g* ∈ *M* of *k*.*i*, we impose that *r*_*eag*_ ≤ *r*_*ebg*_ + 2(*y*_*ak*. *i*_ + *y*_*bk*. *i*_), *r*_*ebg*_ ≤ *r*_*eag*_ + 2(*y*_*ak*. *i*_ + *y*_*bk*. *i*_). These constraints ensure that *r*_*eag*_ = *r*_*ebg*_ for a gene *g* common for *a′*(*z*) and *b′*(*z*).

For each edge *e* and gene *k.j* from *M*, we define the Boolean variable *f*_*ek.j*_ to indicate whether the gene *k.j* is common for *a′*(*z*) and *b′*(*z*). Specifically, for an inner edge *e* we impose that

$$ {f}_{ek.j}\ge 1-{y}_{ak.j}-{y}_{bk.j},{f}_{ek.j}\le 1-{y}_{ak.j},{f}_{ek.j}\le 1-{y}_{bk.j}; $$

while for a leaf edge, the variable *y*_*bk.j*_ is replaced with $1-\sum \limits_i{z}_{bkij}$yielding:

$$ {f}_{ek.j}\ge \sum \limits_i{z}_{bkij}-{y}_{ak.j},{f}_{ek.j}\le \sum \limits_i{z}_{bkij}. $$

For each extremity *g* of gene *k.i* from *M*, we define the integer variable *l*_*eg*_, which equals *r*_*eag*_ if *g* is an extremity of a common gene in *a′*(*z*) and *b′*(*z*), or equals 0 otherwise. The corresponding constraints are as follows:

$$ -{f}_{ek.i}\le {l}_{eg}\le {f}_{ek.i},{l}_{eg}\le {r}_{eag}+2\left(1-{f}_{ek.i}\right),{r}_{eag}\le {l}_{eg}+2\left(1-{f}_{ek.i}\right). $$

Now the statement that ${C_2}^{\prime }=\sum \limits_g{r}_g-\sum \limits_g{l}_g$ for any *e* ∈ *T* is proved in the same manner as in the distance problem.

3) On each edge *e* ∈ *T*, where *e* = (*a*,*b*), each of the first two parentheses in the definition *F′* equals the number of common genes in *a′*(*z*) and *b′*(*z*) counted once plus the total number of special genes in the same structures; this sum will be referred to as *X*. Indeed, the values of ${c}_e-\sum \limits_{k,i}{y}_{ak.i}$ and $2\cdot \mid M\mid -\sum \limits_{k,i}{y}_{ak.i}-\sum \limits_{k,i}{y}_{bk.i}$ equal to the total number of all genes in *a* and *b*. These values minus $\sum \limits_{k,j}{f}_{k.j}$, the number of common genes counted once, gives *X*.

Let Ψ(*e*, *x*, *y*, z) be equal to *C*_0_ + *n* + *s*_*a*_ + *s*_*b*_ in Φ from Section 2. Here, Ψ is actually considered on the edge *e* = (*a*,*b*), and the summands are defined as in Section 2. We obtain *X* − Ψ = *С*_3_ − *C*_0_, where *С*_3_ is the total number of genes in special chromosomes in *a′*(*z*) and *b′*(*z*), and *C*_0_ is the total number of special chromosomes in *a′*(*z*) and *b′*(*z*). We define that $E=\sum \limits_{e\in T}\left({\mathrm{C}}_3-{C}_0\right)(e)$. For any arrangement *A* and the initial numberings, *E*(*A*) is defined analogously.

Theorem 2 states that our reduction algorithm upon the condition (*) is *exact.* To this end, let us introduce the definitions. Assumed that the arguments (*x*,*y*,*z*,*t*,*f*,*u*,*p*,*r*,*l*) of the function *F′ extend* the arguments (*x*,*y*,*z*) of the function F*, if the variable *t* for each *e* defines new adjacencies in *a* ^*+*^(*z*,*t*) and *b* ^*+*^(*z*,*t*) such that the distance between the structures is minimum, and other variables are defined through *a′*(*z*), *b′*(*z*), and *G″* such that the above constraints as well as the equalities ${C_1}^{\prime }=\sum \limits_s{p}_s$ and ${C_2}^{\prime }=\sum \limits_g{r}_g-\sum \limits_g{l}_g$ are satisfied for each edge *e*. Clearly, there is an extension for each arrangement *A =* (*x*,*y*,*z*); any of them is denoted as *A*_+_. Recall that an arrangement *A* defines structures *a* and *b* at the ends of the edge *e* = (*a*,*b*).

#### Theorem 2

Upon (*), the minimum values of functions F*(*A*) and *F′*(*x*,*y*,*z*,*t*,*f*,*u*,*p*,*r*,*l*) are equal. Otherwise, the difference between the minimum values is not greater than the total quantity of special chromosomes in the minimum point of *F′*.

#### Lemma

For any structures *a′*(*z*) and *b′*(*z*) we have *Q*_2_ = *Q*_1_ + *C*_3_ where *Q*_1_ and *Q*_2_ are the maximal values of *C*_1_ + 0.5 ⋅ *C*_2_ and *C*_1_^′^ + 0.5 ⋅ *C*_2_^′^, respectively.

#### Proof of lemma

Let the maximums of *Q*_1_ and *Q*_2_ be reached at the points *t*_0_ and *t′*_0_, respectively. We can add to the structures *a″*(*z*,*t*_0_) and *b″*(*z*,*t*_0_) the removed special chromosomes and new chromosomes that are identical to these special chromosomes. Respectively, *C*_3_ cycles of length 2 are added to the breakpoint graph *a″*(*z*,*t*_0_) + *b″*(*z*,*t*_0_). Thus, *Q*_2_ ≥ *Q*_1_ + *C*_3_.

To prove the inverse relation let us consider the distance *d* between *a′*(*z*) and *b′*(*z*). As we know *d* = *C*_0_ + *n* + *s*_*a*_ + *s*_*b*_ − *Q*_1_. On the other hand, following [12] it is easy to verify that $d\le {C}_0^{\prime }+n+{s}_a+{s}_b+{C}_3-{Q}_2$ where ${C}_0^{\prime }$ is the quantity of new chromosomes that remain unchanged under a transformation of ${a}^{+}\left(z,{t}_0^{\prime}\right)$ into ${b}^{+}\left(z,{t}_0^{\prime}\right)$. Evidently ${C}_0^{\prime}\le {C}_0$. Thus, *Q*_2_ ≤ *Q*_1_ + *C*_3_. □.

#### Proof of theorem 2

For any arrangement *A* and edge *e* = (*a*, *b*) ∈ *T*, it is valid that ${F}^{\ast}\left(\mathrm{A}\right)=\sum \limits_{e\in T}\Phi \left(e,{t}_0\right)$, where Φ(*a*, *b*, *t*) = *C*_0_ + *n* + *s*_*a*_ + *s*_*b*_ − *C*_1_ − 0.5 ⋅ *C*_2_ and *C*_0_, *n*, *s*_*a*_, *s*_*b*_, *C*_1_, *C*_2_ are defined as in Section 2; *t*_0_ is also defined there. By the Lemma we have on each *T*-edge *e* that *C*_1_^′^ + 0.5 ⋅ *C*_2_^′^ = *C*_1_ + 0.5 ⋅ *C*_2_ + *C*_3_. This and item 3 imply that

$$ {F}^{\prime}\left({A}_{+}\right)={F}^{\ast }(A)+E(A)-{C}_3(A)={F}^{\ast }(A)-{C}_0(A). $$

It follows from items 1–2 that any minimum point of the function *F′* is an extension of its coordinates *x,y,z*. Let *A*_+_ be a minimum point of the *F′*. If the condition (*) is satisfied for it, then *E*(*A*) = 0; *A* is the minimum arrangement since *C*_0_(*A*) ≥ 0 for any *A*. If (*) is not satisfied, let *A*_+_ be the point of minimum of *F′*, A* be a minimum arrangement. Then

$$ {F}^{\prime}\left({A}_{+}\right)={F}^{\ast }(A)-{C}_0(A)\ge {F}^{\ast}\left({A}^{\ast}\right)-{C}_0(A),{F}^{\prime}\left({A}_{+}\right)\le {F}^{\prime}\left(A{\ast}_{+}\right)\le {F}^{\ast}\left({A}^{\ast}\right).\square $$

The constants 2∙*|M|* and *c*_*e*_ can be omitted in the minimization.

Notice that the condition (*) limits special (broadly speaking, circular) chromosomes in the structures, i.e., limits the relationship between the parental structure and its direct descendants. Our computer experiments (data not shown) have demonstrated that the solution of the second problem with F* using a heuristic algorithm (described in [17]) differed little from that with *F′* using ILP. Indeed, the evolutionary scenario for mitochondrial chromosome structures generated by the heuristic algorithm in [17] included no special chromosomes.

Clearly, the number of variables and constraints in it cubically depend on the size of the initial data.

### Examples for the reconstruction problem on synthetic data

#### Example 1

Let us consider a tree ((*c*, *d*),(*e*, *f*)) with four leaf structures and three genes in each structure distributed among circular chromosomes: structure *c*, (1, 2, −1); *d*, (1, 1, −2); *e*, (2, 1, −1); *f*, (1, 1, 2). Other designations in all examples are as in Section 2.2.

The initial numberings are as follows: *c*, (1.1, 2, −1.2); *d*, (1.1, 1.2, −2); *e*, (2, 1.1, −1.2); *f*, (1.1, 1.2, 2).

The ILP program of the Pulp python package returned the solution with the total number of operations being 3, one in each edge. The result swaps 1.1 and 1.2 in the leaves *e* and *f*; the chromosome (1.1, 1.2, 2) appears in the root; (1.1, 2, −1.2) is ancestral for the nodes *c* and *d* and (1.1, 2, 1.2) is ancestral for the nodes *e* and *f*. The program execution time was about 13 h.

#### Example 2

Let us consider the same tree with five genes in each leaf structure distributed among linear chromosomes: structure *c*, [2, 3, −4, 1], [1]; *d*, [3, −4, 1], [1, 2]; *e*, [1, −2, 3], [1, 4]; and *f*, [1, −2, 3, 4], [1].

The initial numberings is as follows: the paralogs of gene 1 in each structure have the name 1.1 in the first chromosome and 1.2 in the second chromosome.

The ILP program of the Pulp python package returned the solution with the total number of operations being 6, one in each edge. The result swaps 1.1 and 1.2 in the leaves *c* and *d*; the chromosome [1.1, 2, 3, 4, 1.2] appears in the root; [1.1, 2, 3, −4, 1.2] is ancestral for the nodes *c* and *d* and [1.1, −2, 3, 4, 1.2] is ancestral for the nodes *e* and *f*. The program execution time was about 20 h.

### Examples for the reconstruction problem on biological data

The orthologs of plastid and mitochondrial proteins were obtained using our algorithm and databases available at http://lab6.iitp.ru/ppc/ and http://lab6.iitp.ru/mpc/. The mitochondrial, plastid, and bacterial chromosome structures were extracted from genome annotations in GenBank by our script.

#### Example 1

Let us consider the example from [17], specifically, the tree given in ([17], Figure 4) and the mitochondrial chromosome structures in its leaves listed in ([17], Table 3); which are also given in Table 1 where they are marked by (*l*) after the species name. The mitochondrial chromosomes belong to the sporozoan class Aconoidasida. The ILP program of the package of Joint Supercomputer Center of the Russian Academy of Sciences (http://www.jscc.ru/eng/index.shtml) returned the solution specified in other lines of Table 1. The program execution time was about 2 days. The resulting reconstruction of the mitochondrial chromosome structures is slightly different from that obtained in ([17], Table 3) using the heuristic algorithm in [17]. The result is close to those obtained in [17]. Specifically, the gene *ls*2 encoding a fragment of the large subunit ribosomal RNA becomes in the inner nodes the separate linear chromosome which likely reflects frequent relocations of the fragment. Although ribosomal RNA genes are rarely fragmented, it is arguable that the small fragments can be highly mobile in this case. The tree generated using protein alignments in apicomplexan parasites [26] is in good agreement with the chromosome structure tree.

**Table 1:** Reconstruction obtained by reduction to ILP for mitochondrial chromosome structures in sporozoan class Aconoidasida. The data in the tree leaves are in the lines marked by (*l*) after the species name. It was obtained from genomes represented in GenBank

*Plasmodium fragile – Babesia bovis*	*ls5 ls6 ls2 (L) ss4 ss6 ls7 ss3 ls3 ls9 ss2 ls4 *cox3 ls8 ss5 ss1 cox1 cytb ls1 (C)
*Theileria annulata – Babesia bovis*	cox1 *cox3 ls1 *ls3 *cytb *ls5 ls4 (L)
*Theileria annulata – Theileria parva*	cox1 *cox3 ls1 *ls3 *cytb *ls5 ls4 (L)
*Theileria annulata* (*l*)	cox1 *cox3 ls1 *ls3 *cytb *ls5 ls4 (L)
*Theileria parva* (*l*)	cox1 *cox3 ls1 *ls3 *ls2 *cytb *ls5 ls4 (L)
*Babesia bovis* (*l*)	cox1 *cox3 ls1 *ls2 *ls3 *cytb *ls4 ls5 (L)
*Plasmodium fragile – Plasmodium berghei*	ss4 ss6 ls7 ss3 ls3 ls9 ss2 ls4 *cox3 ls8 ss5 ss1 cox1 cytb ls1 (C) ls2 (L)
*Plasmodium juxtanucleare – Plasmodium berghei*	ls1 ss4 ss6 ls7 ls6 ss3 ls3 ls9 ss2 ls4 ls5 *cox3 ls8 ss5 ss1 cox1 cytb ls2 (L)
*Plasmodium juxtanucleare – Leucocytozoon sabrazesi*	ls1 ss4 ss6 ls7 ls6 ss3 ls3 ls9 ss2 ls4 ls5 *cox3 ls8 ss5 ss1 cox1 cytb ls2 (L)
*Plasmodium juxtanucleare – Plasmodium gallinaceum*	ls1 ss4 ss6 ls7 ls6 ss3 ls3 ls9 ss2 ls4 ls5 *cox3 ls8 ss5 ss1 cox1 cytb ls2 (L)
*Plasmodium juxtanucleare* (*l*)	ls1 ss4 ss6 ls7 ls6 ss3 ls3 ls9 ss2 ls4 ls5 *cox3 ls8 ss5 ss1 cox1 cytb ls2 (L)
*Plasmodium gallinaceum* (*l*)	ls1 ss4 ss6 ls7 ls6 ss3 ls3 ls9 ss2 ls4 ls5 *cox3 ls8 ss5 ss1 cox1 cytb ls2 (L)
*Leucocytozoon sabrazesi* (*l*)	ls1 ss4 ss6 ls7 ls6 ss3 ls3 ls9 ss2 ls4 ls5 *cox3 ls8 ss5 ss1 cox1 cytb ls2 (L)
*Plasmodium berghei* (*l*)	ls1 ss4 ss6 ls7 ls6 ss3 ls3 ls9 ss2 ls4 ls5 *cox3 ls8 ss5 ss1 cox1 cytb ls2 (L)
*Plasmodium fragile – Plasmodium relictum*	ss4 ss6 ls7 ss3 ls3 ls9 ss2 ls4 *cox3 ls8 ss5 ss1 cox1 cytb ls1 (C) ls2 (L)
*Plasmodium reichenowi – Plasmodium relictum*	ss4 ss6 ls7 ss3 ls3 ls9 ss2 ls4 *cox3 ls8 ss5 ss1 cox1 cytb ls1 (C) ls2 (L)
*Plasmodium floridense – Plasmodium relictum*	ls7 ss3 ls3 ls9 ss2 ls4 *cox3 ls8 ss5 ss1 cox1 cytb ls1 ss6 (C)
*Plasmodium floridense* (*l*)	ss3 ls3 ls9 ss2 ls4 *cox3 ls8 ss5 ss1 cox1 cytb ls1 ss4 ss6 ls7 (L)
*Plasmodium relictum* (*l*)	ss3 ls3 ls9 ss2 ls4 *cox3 ls8 ss5 ss1 cox1 cytb ls1 ss4 ss6 ls7 (C)
*Plasmodium reichenowi – Plasmodium mexicanum*	ss3 ls3 ls9 ss2 *cox3 ls8 ss5 ss1 cox1 cytb ls1 ss4 ss6 ls7 (L)
*Plasmodium reichenowi – Plasmodium falciparum*	ss3 ls3 ls9 ss2 *cox3 ls8 ss5 ss1 cox1 cytb ls1 ss4 ss6 ls7 (L)
*Plasmodium reichenowi* (*l*)	ss3 ls3 ls9 ss2 *cox3 ls8 ss5 ss1 cox1 cytb ls1 ss4 ss6 ls7 (L)
*Plasmodium falciparum* (*l*)	ss3 ls3 ls9 ss2 *cox3 ls8 ss5 ss1 cox1 cytb ls1 ss4 ss6 ls7 (L)
*Plasmodium mexicanum* (*l*)	ss3 ls3 ls9 ss2 *cox3 ls8 ss5 ss1 cox1 cytb ls1 ss4 ss6 ls7 (L)
*Plasmodium fragile – Plasmodium simium*	ss4 ss6 ls7 ss3 ls3 ls9 ss2 ls4 *cox3 ls8 ss5 ss1 cox1 cytb ls1 (C)
*Plasmodium fragile – Leucocytozoon fringillinarum*	ss3 ls3 ls9 ss2 ls4 *cox3 ls8 ss5 ss1 cox1 cytb ls1 ss4 ss6 ls7 (L)
*Plasmodium fragile – Plasmodium vivax*	ls1 ss6 ls7 ss3 ls3 ls9 ss2 ls4 *cox3 ls8 ss5 ss1 cox1 cytb (C)
*Plasmodium fragile – Plasmodium knowlesi*	ls1 ss6 ls7 ss3 ls3 ls9 ss2 ls4 *cox3 ls8 ss5 ss1 cox1 cytb (C)
*Plasmodium fragile – Leucocytozoon majoris*	ls1 ss6 ls7 ss3 ls3 ls9 ss2 ls4 *cox3 ls8 ss5 ss1 cox1 cytb (C)
*Plasmodium fragile* (*l*)	ls1 ss6 ls7 ss3 ls3 ls9 ss2 ls4 *cox3 ls8 ss5 ss1 cox1 cytb (C)
*Leucocytozoon majoris* (*l*)	ss3 ls3 ls9 ss2 ls4 *cox3 ls8 ss5 ss1 cox1 cytb ls1 ss6 ls7 (C)
*Plasmodium knowlesi* (*l*)	ls1 ss6 ls7 ss3 ls3 ls9 ss2 ls4 *cox3 ls8 ss5 ss1 cox1 cytb (C)
*Plasmodium vivax* (*l*)	ls1 ss6 ls7 ss3 ls3 ls9 ss2 ls4 *cox3 ls8 ss5 ss1 cox1 cytb (C)
*Leucocytozoon fringillinarum* (*l*)	ss3 ls3 ls9 ss2 ls4 *cox3 ls8 ss5 ss1 cox1 cytb ls1 ss6 ls7 ss4 (C)
*Plasmodium simium* (*l*)	ls1 ss6 ls7 ss3 ls3 ls9 ss2 ls4 *cox3 cox1 cytb ls8 ss5 ss1 (C)

If a structure has two chromosomes, they are given on separate lines. Circular and linear chromosomes are marked by (C) and (L), respectively. The symbol * means the complementary chain

#### Example 2

Let us exemplify the reconstruction for plastid chromosome structures with paralogs in brown algae. They are also given in Table 2 marked by (*l*) after the species name. The following chromosome structure tree was built: (Ectocarpus_siliculosus, (Fucus_vesiculosus, Saccharina_japonica)). The tree that was generated using highly conserved elements identified in the complete plastid genomes of all considered species [27] is in good agreement with the chromosome structure one. The reconstruction result is presented in Table 2. The program execution time was about 5 days.

**Table 2:** Reconstruction obtained by reduction to ILP for plastid chromosome structures with paralogs in brown algae

*Ectocarpus siliculosus* (*l*)	rpl32_1 rpl21_1 *rps4 *rps16 *rps1 rpl9 rpl11 rpl1 rpl12 *rps10 *tufa *rps7 *rps12 *rpl31 *rps9 *rpl13 *rpoa *rps11 *rps13 *rpl36 *rps5 *rpl18 *rpl6 *rps8 *rpl5 *rpl24 *rpl14 *rps17 *rpl29 *rpl16 *rps3 *rpl22 *rps19 *rpl2 *rpl23 *rpl4 *rpl3 *rpl21_2 *rpl32_2 *rpl35 rpl20 *rpl19 rpl27 rpl34 rps20 rpob rpoc1 rpoc2 rps2 rps14 *rps18 *rpl33 clpc rbcl (C)
*Fucus vesiculosus* (*l*)	*rpl19 rpl27 rpl34 rps20 rpob rpoc1 rpoc2 rps2 rpl35 rpl20 rbcl rps14 *clpc rpl33 rps18 *rpl32_2 rps16 rps4 rps1 rpl9 rpl11 rpl1 rpl12 *rps10 *tufa *rps7 *rps12 *rpl31 *rps9 *rpl13 *rpoa *rps11 *rps13 *rpl36 *rps5 *rpl18 *rpl6 *rps8 *rpl5 *rpl24 *rpl14 *rps17 *rpl29 *rpl16 *rps3 *rpl22 *rps19 *rpl2 *rpl23 *rpl4 *rpl3 *rpl21_2 (C)
*Saccharina japonica* (*l*)	*rps2 *rpoc2 *rpoc1 *rpob *rps20 *rpl34 *rpl27 rpl19 rpl35 rpl20 rbcl rps14 *rps18 *rpl33 clpc rpl32_1 rpl21_1 rpl3 rpl4 rpl23 rpl2 rps19 rpl22 rps3 rpl16 rpl29 rps17 rpl14 rpl24 rpl5 rps8 rpl6 rpl18 rps5 rpl36 rps13 rps11 rpoa rpl13 rps9 rpl31 rps12 rps7 tufa rps10 *rpl12 *rpl1 *rpl11 *rpl9 rps1 *rps4 *rps16 (C)
Inner non-root node	*rpl19 rpl27 rpl34 rps20 rpob rpoc1 rpoc2 rps2 rpl35 rpl20 rbcl rps14 rpl32_2 *rps18 *rpl33 clpc rpl32_1 rpl21_1 *rps4 *rps16 *rps1 rpl9 rpl11 rpl1 rpl12 *rps10 *tufa *rps7 *rps12 *rpl31 *rps9 *rpl13 *rpoa *rps11 *rps13 *rpl36 *rps5 *rpl18 *rpl6 *rps8 *rpl5 *rpl24 *rpl14 *rps17 *rpl29 *rpl16 *rps3 *rpl22 *rps19 *rpl2 *rpl23 *rpl4 *rpl3 *rpl21_2 (C)
Tree root	rpl32_1 rpl21_1 *rps4 *rps16 *rps1 rpl9 rpl11 rpl1 rpl12 *rps10 *tufa *rps7 *rps12 *rpl31 *rps9 *rpl13 *rpoa *rps11 *rps13 *rpl36 *rps5 *rpl18 *rpl6 *rps8 *rpl5 *rpl24 *rpl14 *rps17 *rpl29 *rpl16 *rps3 *rpl22 *rps19 *rpl2 *rpl23 *rpl4 *rpl3 *rpl21_2 *rpl19 rpl27 rpl34 rps20 rpob rpoc1 rpoc2 rps2 rps14 rpl32_2 *rps18 *rpl33 clpc rpl35 rpl20 rbcl (C)

Paralog numbers are given after the underscore. For other designations, see Table 1

#### Example 3

**Fig. 2:**
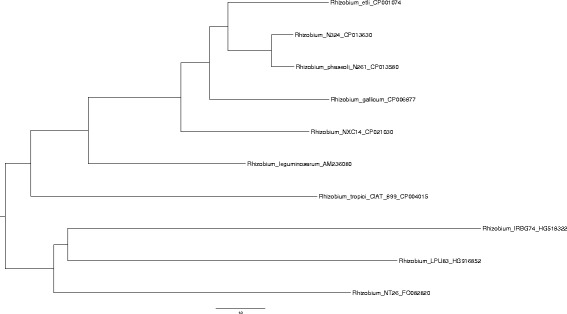
Tree of chromosomal structures of *Rhizobium* spp. generated using the chromosome structures given in Table 3 in the lines marked by (*l*) after the species name. The reconstruction result is presented in the other lines of the same Table 3

Let us exemplify the reconstruction for chromosome structures with paralogs from *Rhizobium* spp. The corresponding tree generated here using chromosome structures is given in Table 3 in the lines marked by (*l*) is shown in Fig. 2. The reconstruction result is presented in other lines of Table 3. The program execution time was about 11 days.

**Table 3:** Reconstruction obtained by reduction to ILP for chromosome structures in *Rhizobium* spp.

*Rhizobium_N324_CP013630* (*l*)	*rpsA *rpsO rplT rpsT rpoN rpoE_1 rpsU_1 rpoZ *rplI *rpsR *rpsF *rpsI *rplM rplK rplA rplJ rplL rpoB rpoC rpsL rpsG rpsJ rplC rplD rplW rplB rpsS rplV rpsC rplP rpsQ rplN rplX rplE rpsN rpsH rplF rplR rpsE rplO rpsM rpsK rpoA rplQ rpsB *rpsD rpoE_2 rplY rpoH_1 rpsU_2 rpoE_3 rpsP rplS *rpoH_2 rplU (C)
*Rhizobium_phaseoli_straiNN261_CP013580* (*l*)	*rpsA *rpsO rplT rpsT rpoN rpoE_1 rpsU_1 rpoZ *rplI *rpsR *rpsF *rpsI *rplM rplK rplA rplJ rplL rpoB rpoC rpsL rpsG rpsJ rplC rplD rplW rplB rpsS rplV rpsC rplP rpsQ rplN rplX rplE rpsN rpsH rplF rplR rpsE rplO rpsM rpsK rpoA rplQ rpsB *rpsD rpoE2 rplY rpoH_1 rpsU_2 rpoE_3 rpsP rplS rpoH_2 rplU (C)
*Rhizobium_N324_CP013630 – Rhizobium_phaseoli_N261_CP013580*	*rpsA *rpsO rplT rpsT rpoN rpoE_1 rpsU_1 rpoZ *rplI *rpsR *rpsF *rpsI *rplM rplK rplA rplJ rplL rpoB rpoC rpsL rpsG rpsJ rplC rplD rplW rplB rpsS rplV rpsC rplP rpsQ rplN rplX rplE rpsN rpsH rplF rplR rpsE rplO rpsM rpsK rpoA rplQ rpsB *rpsD rpoE2 rplY rpoH_1 rpsU_2 rpoE_3 rpsP rplS rpoH_2 rplU (C)
*Rhizobium_etli_CP001074* (*l*)	*rpsA *rpsO rplT rpsT rpoN rpoE_1 rpsU_1 rpoZ *rplI *rpsR *rpsF *rpsI *rplM rplK rplA rplJ rplL rpoB rpoC rpsL rpsG rpsJ rplC rplD rplW rplB rpsS rplV rpsC rplP rpsQ rplN rplX rplE rpsN rpsH rplF rplR rpsE rplO rpsM rpsK rpoA rplQ rpsB *rpsD rpoE2 rplY rpoE_3 *rpoE_4 rpoH_1 rpsU_2 rpsP rplS rpoH_2 rplU (C)
*Rhizobium_etli_CP001074* – *Rhizobium_phaseoli_N261_CP013580*	*rpsA *rpsO rplT rpsT rpoN rpoE_1 rpsU_1 rpoZ *rplI *rpsR *rpsF *rpsI *rplM rplK rplA rplJ rplL rpoB rpoC rpsL rpsG rpsJ rplC rplD rplW rplB rpsS rplV rpsC rplP rpsQ rplN rplX rplE rpsN rpsH rplF rplR rpsE rplO rpsM rpsK rpoA rplQ rpsB *rpsD rpoE_2 rplY rpoH_1 rpsU_2 rpoE_3 rpsP rplS rpoH_2 rplU (C)
*Rhizobium_gallicum_CP006877* (*l*)	*rpsA rpsO rplT rpsT rpoN rpoE_1 rpoZ *rplI *rpsR *rpsF *rpsI *rplM rplK rplA rplJ rplL rpoB rpoC rpsL rpsG rpsJ rplC rplD rplW rplB rpsS rplV rpsC rplP rpsQ rplN rplX rplE rpsN rpsH rplF rplR rpsE rplO rpsM rpsK rpoA rplQ rpsB *rpsD rpoE_2 rplY rpoH_1 *rpsU_1 rpsU_2 rpsP rplS *rpoH_2 rplU (C)
*Rhizobium_etli_CP001074* – *Rhizobium_gallicum_CP006877*	*rpsA *rpsO rplT rpsT rpoN rpoE_1 rpsU_1 rpoZ *rplI *rpsR *rpsF *rpsI *rplM rplK rplA rplJ rplL rpoB rpoC rpsL rpsG rpsJ rplC rplD rplW rplB rpsS rplV rpsC rplP rpsQ rplN rplX rplE rpsN rpsH rplF rplR rpsE rplO rpsM rpsK rpoA rplQ rpsB *rpsD rpoE2 rplY rpoH_1 rpsU_2 rpsP rplS rpoH_2 rplU (C)
*Rhizobium_NXC14_CP021030* (*l*)	*rpsA *rpsO rplT rpsT rpoN rpoE_1 rpoE_3 rpsU_1 rpoZ *rplI *rpsR *rpsF *rpsI *rplM rplK rplA rplJ rplL rpoB rpoC rpsL rpsG rpsJ rplC rplD rplW rplB rpsS rplV rpsC rplP rpsQ rplN rplX rplE rpsN rpsH rplF rplR rpsE rplO rpsM rpsK rpoA rplQ rpsB *rpsD rpoE_2 rplY rpoH_1 rpsU_2 rpsP rplS rpoH_2 rplU (C)
*Rhizobium_etli_CP001074* – *Rhizobium_NXC14_CP021030*	*rpsA *rpsO rplT rpsT rpoN rpoE_1 rpsU_1 rpoZ *rplI *rpsR *rpsF *rpsI *rplM rplK rplA rplJ rplL rpoB rpoC rpsL rpsG rpsJ rplC rplD rplW rplB rpsS rplV rpsC rplP rpsQ rplN rplX rplE rpsN rpsH rplF rplR rpsE rplO rpsM rpsK rpoA rplQ rpsB *rpsD rpoE_2 rplY rpoH_1 rpsU_2 rpsP rplS rpoH_2 rplU (C)
*Rhizobium_leguminosarum_AM236080* (*l*)	*rpsA *rpsO rplT rpsT rpoN rpsU_1 *rplI *rpsR *rpsF *rpsI *rplM rplK rplA rplJ rplL rpoB rpoC rpsL rpsG rpsJ rplC rplD rplW rplB rpsS rplV rpsC rplP rpsQ rplN rplX rplE rpsN rpsH rplF rplR rpsE rplO rpsM rpsK rpoA rplQ rpsB *rpsD *rpoD *rpoZ rpoH_1 rpsU_2 rpoE_3 rplU (C)
*Rhizobium_etli_CP001074* – *Rhizobium_leguminosarum_AM236080*	*rpsA *rpsO rplT rpsT rpoN rpoE_1 rpsU_1 rpoZ *rplI *rpsR *rpsF *rpsI *rplM rplK rplA rplJ rplL rpoB rpoC rpsL rpsG rpsJ rplC rplD rplW rplB rpsS rplV rpsC rplP rpsQ rplN rplX rplE rpsN rpsH rplF rplR rpsE rplO rpsM rpsK rpoA rplQ rpsB *rpsD rpoH_1 rpsU_2 rpoH_2 rplU (C)
*Rhizobium_tropici_CIAT_899_CP004015* (*l*)	*rpsA *rpsO rplT rpsT *rpoN *rplI *rpsR *rpsI *rplM rplK rplA rplL rpoB rpoC rpsL rpsG rpsJ rplC rplD rplW rplB rplV rpsC rplP rpsQ rplN rplX rpsH rplF rplR rpsE rplO rpsM rpsK rpoA rplQ rpsB *rpsD rpoH_1 *rpoH_2 rplU (C)
*Rhizobium_etli_CP001074* – *Rhizobium_tropici_CIAT_899_CP004015*	*rpsA *rpsO rplT rpsT rpoN rpsU1 rpoZ *rplI *rpsR *rpsF *rpsI *rplM rplK rplA rplJ rplL rpoB rpoC rpsL rpsG rpsJ rplC rplD rplW rplB rpsS rplV rpsC rplP rpsQ rplN rplX rplE rpsN rpsH rplF rplR rpsE rplO rpsM rpsK rpoA rplQ rpsB *rpsD rpoH_1 rpsU_2 rpoH_2 rplU (C)
*Rhizobium_IRBG74_HG518322* (*l*)	*rpsO rplT rpsT *rpoN rpoZ *rpsR *rpsF *rpsI *rplM rpsB *rpsD *rplQ *rpoA *rpsK *rpsM *rplO *rpsE *rplR *rplF *rpsH *rpsN *rplE *rplX *rplN *rplP *rpsC *rplV *rpsS *rplB *rplW *rplD *rplC *rpsJ *rpsG *rpsL *rpoC *rpoB *rplL *rplJ *rplA *rplK *rpoD rplY rpoH_1 rpsP rplS *rplU (C) rpsU_1 *rpsU_2 rpsA (L)
*Rhizobium_LPU83_HG916852* (*l*)	rpsO rpsA rplT rpsT rpoN rpoZ *rplI *rpsR *rpsF rplK rplA rplJ rplL rpoB rpoC rpsL rpsG rpsJ rplC rplD rplW rplB rpsS rplV rpsC rplP rpsQ rplN rplX rplE rpsN rpsH rplF rplR rpsE rplO rpsM rpsK rpoA rplQ *rpsI *rplM rpsD *rpsB *rpoD rplY rpoH_1 rpsU_1 *rpsU_2 rpsU_3 rpsP rplS rpoH2 *rplU (C)
*Rhizobium_IRBG74_HG518322* – *Rhizobium_LPU83_HG916852*	rpsO rpsA rplT rpsT rpoN rpoZ *rplI *rpsR *rpsF *rpsI *rplM rplK rplA rplJ rplL rpoB rpoC rpsL rpsG rpsJ rplC rplD rplW rplB rpsS rplV rpsC rplP rpsQ rplN rplX rplE rpsN rpsH rplF rplR rpsE rplO rpsM rpsK rpoA rplQ rpsD *rpsB *rpoD rplY rpoH_1 rpsU_1 *rpsU_2 rpsU_3 rpsP rplS rpoH_2 *rplU (C)
*Rhizobium_NT26_FO082820* (*l*)	rpsO rpsA rplT *rpoN rpoZ *rplI *rpsR *rpsF *rpsI *rplM rplK rplA rplJ rplL rpoB rpoC rpsL rpsG rpsJ rplC rplD rplW rplB rpsS rplV rpsC rplP rplN rplX rplE rpsN rpsH rplF rplR rpsE rplO rpsM rpsK rpoA rplQ rpsB rpsU_2 *rpsD *rpoD rplY rpoH_1 rpsU_1 rplU rpsP rplS *rpoH_2 (C)
*Rhizobium_IRBG74_HG518322* – *Rhizobium_NT26_FO082820*	rpsO rpsA rplT rpsT rpoN rpoZ *rplI *rpsR *rpsF *rpsI *rplM rplK rplA rplJ rplL rpoB rpoC rpsL rpsG rpsJ rplC rplD rplW rplB rpsS rplV rpsC rplP rpsQ rplN rplX rplE rpsN rpsH rplF rplR rpsE rplO rpsM rpsK rpoA rplQ rpsB *rpsD *rpoD rplY rpoH_1 rpsU_1 rpsP rplS rpoH_2 *rplU (C)
Tree root	rpsO rpsA rplT rpsT rpoN rpsU_2 rpoZ *rplI *rpsR *rpsF *rpsI *rplM rplK rplA rplJ rplL rpoB rpoC rpsL rpsG rpsJ rplC rplD rplW rplB rpsS rplV rpsC rplP rpsQ rplN rplX rplE rpsN rpsH rplF rplR rpsE rplO rpsM rpsK rpoA rplQ rpsB *rpsD *rpoD rplY rpoH_1 rpsU_1 rpsP rplS rpoH_2 rplU (C)

For other designations, see Tables 1 and 2

## Solution of the problem of optimal arrangement of contigs

Let us apply the developed approach to the *contig* problem, optimal genome assembly from contigs. The biological significance of the problem is discussed in [28].

### Contig problem statement

**Fig. 3:**
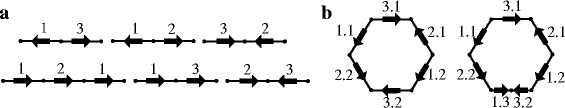
**a** Given sets *a* and *b* composed of three contigs each. **b** Problem solution: the minimum cycles for (**a**) (left) and (**b**) (right)

Sequencing results in a set *a* of contigs (or scaffolds, or sequences of a higher level, etc.), each of which includes several genes with their own direction of transcription. Here, a contig is considered as a *path* of genes each with a name not necessarily unique (paralogs) and a direction (Fig. 3a). Therefore, *a* is a structure comprised of paths. Two contigs can be concatenated in four ways considering that a contig is a double-stranded DNA region with undefined beginning and end. A set of contigs can be concatenated into a long path or cycle; these variants are essentially equivalent, and we will consider the second one as in [28]. It is convenient to consider that each contig ends with an extremity of one of its genes.

The *contig* problem is as follows. We are given two sets *a* and *b* of contigs, and it is required to concatenate contigs from *a* into one cycle and contigs from *b* into another cycle, and simultaneously find paralog numberings (see Section 1.3) with the minimum distance between the cycles without paralogs (Fig. 3b). Naturally, these cycles are considered as structures comprised of sole cycle each. Similarly to the solution below, a more general case is considered when contigs from *a* and, similarly, from *b* are concatenated into structures of another fixed shape.

An almost linear (to be precise, *n* ⋅ *f*(*n*), where *f*(*n*) is the inverse Ackermann′s function) algorithm was proposed in [28]; it exactly solves the contig problem on the condition of equal gene content of two sets of contigs (*n* genes in each) and without paralogs. Below is the solution of the problem with this *condition released* based on its reduction to ILP. The presence of paralogs makes the problem NP-hard. In addition, the solution in [28] relies on the algebraic theory of permutation groups, which absolutely differs from our approach and relies on a different distance. Specifically, in the case of equal gene content, our distance (in the terms specified in Sections 1–2) equals *n* − *C*_1_ − 0.5*C*_2_, where *C*_1_ is the quantity of cycles and *C*_2_ is the quantity of even paths in the breakpoint graph; while the distance used in [28] can be calculated using the same expression but with *C*_2_ being the quantity of all paths. We fix arbitrary initial numberings of paralogs, and the structures *a* and *b* with fixed numberings are denoted as *a′* and *b′*.

In the next section the reduction of the contig problem to ILP is presented, which simultaneously determines the numberings and the above mentioned two cycles with the minimum distance between them. The resulting cycles will be referred to as *minimum*. Our solution for two sets of contigs can be similarly extended to an arbitrary number of sets. In this problem, it is important to discriminate between *outer* and *inner adjacencies*. The former merge the extremities of contigs, while the latter merge the extremities of genes within contigs. The contig problem concerns the selection of outer adjacencies that transform two given sets of contigs into two cycles with the minimum distance between them while the inner adjacencies remain unaltered. However, calculation of the distance between cycles includes the variation of inner adjacencies. The distance calculation allows all six operations mentioned in Section 1. Thus, both adjacency types are used altogether.

### Solution of the contig problem

A reduction algorithm for the contig problem to ILP is described below.

For each pair *s* = (*g*_1_,*g*_2_) of extremities of different contigs in *a′*, we define the Boolean variable *t*_*as*_. It equals 1 if *g*_1_ and *g*_2_ form the outer adjacency; otherwise *t*_*as*_ = 0. Similarly for *b′*. The usual constraints ensure that each contig extremity is merged with exactly one contig extremity.

For each ordered pair *d* = (*c*_1_,*c*_2_) of different contigs from either given set *a′* or *b′*, we define the Boolean variable *v*_*d*_ to indicate whether the contig *c*_2_ is concatenated with *c*_1_ and is placed after it; the set of all values *v*_*d*_ = 1 consistently determines the clockwise order on a required cycle. First, the usual constraints ensure that each contig is concatenated with exactly one contig on either side. The constraint *v*_*d*_ ≤ *t*_*s*1_ + *t*_*s*2_ + *t*_*s*3_ + *t*_*s*4_ (for pairs *s*_1_, *s*_2_, *s*_3_, *s*_4_ of extremities of the contigs *c*_1_ and *c*_2_) provides the relation between the order and the outer adjacencies. Let us define the integer (non-Boolean) variables *w*_*ac*_ and *w*_*bc*_, where *c* runs over all contigs in *a′* or *b′* and 1 ≤ *w*_*c*_ ≤ *N* (*N* is the quantity of contigs in the corresponding set). The variable *w*_*ac*_ numbers all contigs in strictly increasing order according to their position in the cycle, this order is violated only in the last contig. Similarly for *w*_*bc*_. For each ordered pair *d* = (*c*_1_,*c*_2_) of different contigs from either set *a′* or *b′*, we define the Boolean variable *r*_*d*_ to indicate the contig where this order is violated. It equals 1 if *v*_*d*_ = 1 and *w*_*c*2_ ≤ *w*_*c*1_, or 0 otherwise. The corresponding constraints are as follows: *r*_*d*_ ≤ *v*_*d*_, *Nr*_*d*_ ≤ *N* − (*w*_*c*2_ − *w*_*c*1_), *Nr*_*d*_ ≥ *w*_*c*1_ − *w*_*c*2_ + 1. Finally, $\sum \limits_d{r}_d=1$ ensures that all contigs of the set are concatenated into a single circular chromosome, where they are numbered by the variable *w* in strictly increasing order.

The further reduction of the contig problem to ILP corresponds to the layout in [17] (or the general case of such reduction was considered in Section 2 above). Namely, let us introduce the Boolean variable *z*_*kij*_, where *z*_*kij*_ = 1 if the gene *k.i* in *a′* corresponds to the gene *k.j* in *b′*; otherwise *z*_*kij*_ = 0. The standard constraints ensure that *z*_*kij*_ defines a partial bijection of *k*-paralogs. If *z*_*kij*_ = 1, the gene *k.j* in *b′* is renamed to *k.i* and becomes synonymous to *k.i* in *a′*, after which the genes in the *z*-bijection are arbitrarily numbered to keep the structures numbered. Structures resulting from such renumbering in *b′*, *are denoted as a′*(*z*) and *b′*(*z*). Adjacencies of the contigs in the cycle are defined by the variable *t* as in Section 2. The resulting two cycles will be referred to as *a′*(*z*,*t*) and *b′*(*z*,*t*). Notice that these structures have unequal gene content. Let us define *G* = *a′*(*z,t*) + *b′*(*z,t*); this breakpoint graph is composed of cycles. It is close to *G′* in Section 2, although equal gene contents were considered there. Let us focus on the differences of the current procedure from that in [17] remembering the presence of outer adjacencies.

1. The quantity *B* of blocks in *G* is expressed by the variable *x*_*as*_ for each *s*, where *s* is an inner adjacency or a pair of contigs extremities in *a′*. It equals 1 if *s* is a boundary of a block in *a′*(*z,t*), and 0 otherwise. Similarly for *x*_*bs*_ and *b′*(*z,t*). Specifically, each *s* in *a′*, is imposed the constraint ${x}_{as}\ge \sum \limits_j{z}_{ki1j}-\sum \limits_j{z}_{li2j}+\left({t}_s-1\right)$, where *k*.*i*_1_ and *l*.*i*_2_ are genes in *a′* with these extremities. Similarly for *s* in *b′*. For an inner adjacency *s*, the summand *t*_*s*_ – 1 is omitted. Let the objective function be

$$ H=0.5\cdot \sum \limits_s{x}_s+\sum \limits_s{y}_s-\sum \limits_s{p}_s. $$

Thus, $B=0.5\cdot \sum \limits_s{x}_s$ at the minimum point of *H*.

2) The sum *S*_1_ of integer parts of half-lengths of the maximal connected regions of conventional edges in *G* is expressed through the Boolean variables *y*_*as*_ and *y*_*bs*_ for all *s* as in subsection (1). It equals 0 if *s* is a boundary or within a block in *a′*(*z,t*) or *b′*(*z,t*); while for the adjacencies of common genes, *y*_*as*_ and *y*_*bs*_ on the edges of *G* alternate within each such region and equal to zero at the ends of odd regions. Specifically, for each pair *s*_1_ in *a′* and *s*_2_ in *b′*, where gene *k.i* is adjacent to gene *k*_1_.*i*_1_ in *s*_1_ and gene *k.j* is adjacent to gene *k*_2_.*i*_2_ in *s*_2_ we impose that

$$ {y}_{as1}+{y}_{bs2}\ge {z}_{kij}+\sum \limits_j{z}_{k1i1j}+\sum \limits_j{z}_{k2 ji2}-2+\left({t}_{as1}-1\right)+\left({t}_{bs2}-1\right), $$

where the summands *t*_*as*1_ *–*1 and *t*_*bs*2_ *–*1 are omitted for inner adjacencies *s*1 and *s*2, respectively. It implies that *y*_*s*_ cannot equal 0 at both neighboring conventional edges. Consequently, it implies that the minimum quantity of unities on the region is reached for the arrangement where zeros alternate with unities starting with zero. Thus, ${S}_1=\sum \limits_s{y}_s$.

3) The quantity *S*_2_ of cycles in *G* composed of conventional edges is expressed in the variables *u*_*s*_ and *p*_*s*_ for *s* as in subsection (1) (see also section 2 and [17]). For each *s*, we impose that ${u}_s\le {m}_s\sum \limits_j{z}_{kij}$ (for *s* from *a′*) or ${u}_s\le {m}_s\sum \limits_j{z}_{kji}$ (for *s* from *b′*), where *k*.*i* is a gene with an extremity from *s*. For each pair *s* of contig extremities, we impose that *u*_*s*_ ≤ *m*_*s*_ *t*_*s*_. In addition, for each pair *s*1 and *s*2 that include extremities of genes *k*.*i* from *a′* and *k*.*j* from *b′*, we impose that

$$ {u}_{s1}\le {u}_{s2}+{m}_{s1}\left(1-{z}_{kij}\right)+{m}_{s1}\left(1-{t}_{s2}\right),{u}_{s2}\le {u}_{s1}+{m}_{s2}\left(1-{z}_{kij}\right)+{m}_{s2}\left(1-{t}_{s1}\right), $$

where, the summand *m*_*s*2_ (1 – *t*_*s*1_) and *m*_*s*1_ (1 – *t*_*s*2_) are omitted for inner adjacencies *s*1 and *s*2, respectively. Then ${S}_2=\sum \limits_s{p}_s$ at the minimum point. The proof is similar to the proof that the quantity *C*_1_ of cycles in *G′* equals $\sum \limits_s{p}_s$ in Section 2.

Therefore, the minimum value of function *H* equals *B* + *S*_1_ − *S*_2_, which equals the distance between the desired cycles [16]. Indeed, lemma 5 and theorem 6 in [16] suggest that the distance equals *B + S + D–P* where *B* is the quantity of special nodes (that is, blocks) in *G*; *S* equals *S*_1_ plus the quantity *S*_3_ of such odd regions at a boundary of any path minus *S*_2_; *D* is the sum of defects of components in the graph *G*; *P* is the quantity of operations, optimized through the interaction of chains in the graph *G*, [16, item 3.4]. Circular *G* has no paths, hence, *D*, *S*_3_, and *P* equal zero.

Clearly, the number of variables and constraints in it quadratically depend on the size of the data.

### Examples for the contig problem on synthetic data

#### Example 1

We are given two sets, *a* (upper) and *b* (lower), each composed of three contigs (Fig. 3a). The initial numberings are as follows (left to right): *a′*, [1.1, 3.1], [1.2, 2.1], and [3.2, 2.2]; *b′*, [1.1, 2.1, 1.2], [1.3, 3.1], and [2.2, 3.2]. Other designations in all examples are as in Section 2.2. The ILP program of the Pulp python package returned the desired minimum cycles for *a′* and *b′* (on the left and on the right in Fig. 3b, respectively). The program execution time was about 6 h.

#### Example 2

We are given two sets, *a*: [−2,1,3], [5,2,–3], [−2,–4,3], [−5,–4,1], [−1,4] and *b*: [3,–2,–4], [3,–1,4,5], [−1,1], [2,–3,–5], [3,–1,–5], [−4,2]. The ILP program of the Pulp python package returned the following minimum cycles for *a* and *b* (outer adjacencies are indicated by the symbol “|”): *a*, (1.2, 3.2 | –2.3, −4.2, 3.1 | –1.3, 4.3 | 5.1, 2.1, −3.3 | –5.2, −4.1, 1.1 | –2.2) and *b*, (1.2 | 3.2, −2.3, −4.2 | 3.1, −1.3, −5.3 | 3.4, −1.4, 4.3, 5.1 | 2.1, −3.3, −5.2 | –4.1, 2.2 | –1.1). The program execution time was about 11 h.

## Conclusions

Three problems are considered; all assume unequal gene content and the presence of gene paralogs. These problems are: (1) to determine the minimum number of operations required to transform one chromosome structure into another and the corresponding transformation itself including the paralog identification; (2) to reconstruct along a tree the chromosome structures given in its leaves; (3) to find the optimal arrangements for each given set of contigs, which also includes the paralog identification.

We proved that these problems can be reduced to integer linear programming, which allows an efficient algorithm to redefine the problems to implement integer linear programming tools. The results were tested on synthetic and biological samples.

## Abbreviations

DNA: Deoxyribonucleic acid
ILP: Integer linear programming
RNA: Ribonucleic acid
